# The PP2A-2 holoenzyme orchestrates daughter cell emergence during cytokinesis in *Toxoplasma gondii*

**DOI:** 10.1371/journal.ppat.1013475

**Published:** 2025-09-03

**Authors:** Jin-Lei Wang, Meng Wang, Nian-Zhang Zhang, Ting-Ting Li, Jin Gao, Xiao-Jing Wu, Bao-Quan Fu, Hany M. Elsheikha, Xing-Quan Zhu

**Affiliations:** 1 State Key Laboratory for Animal Disease Control and Prevention, College of Veterinary Medicine, Lanzhou University, Lanzhou Veterinary Research Institute, Chinese Academy of Agricultural Sciences, Lanzhou, China; 2 State Key Laboratory for Animal Disease Control and Prevention, Key Laboratory of Veterinary Parasitology of Gansu Province, Lanzhou Veterinary Research Institute, Chinese Academy of Agricultural Sciences, Lanzhou, China; 3 Laboratory of Parasitic Diseases, College of Veterinary Medicine, Shanxi Agricultural University, Taigu, China; 4 Faculty of Medicine and Health Sciences, School of Veterinary Medicine and Science, University of Nottingham, Loughborough, United Kingdom; University of Wisconsin Medical School, UNITED STATES OF AMERICA

## Abstract

*Toxoplasma gondii* is a significant pathogen in both humans and animals, with disease progression driven by the rapid proliferation of its tachyzoite stage. In this study, we identify the PP2A-2 holoenzyme as a key regulator of daughter cell emergence during parasite division. This holoenzyme, likely composed of the regulatory subunit TgPR48 (PP2A-B2), the catalytic subunit PP2A-C2, and the scaffolding subunit PP2A-A2, is essential for proper cytokinesis. Disruption of any single component severely impairs daughter cell separation and emergence. Phosphoproteomic analysis following PP2A-C2 depletion revealed numerous differentially phosphorylated proteins. Among these, DCS1 and DCS2 were prioritized as potential effectors. While phosphomimetic and non-phosphorylatable mutations in DCS1 and DCS2 did not significantly impair their function, depletion of either protein disrupted TgPR48 localization. Interestingly, TgPR48 overexpression partially rescued the phenotypes associated with DCS2 loss, but not DCS1, indicating divergence in their downstream pathways and implicating additional, yet unidentified, substrates. These findings establish PP2A-2–mediated dephosphorylation as a central mechanism in regulating tachyzoite cytokinesis and highlight a promising regulatory axis for therapeutic intervention against *T. gondii*.

## Author summary

Daughter cell emergence is a critical step in the lytic cycle of the apicomplexan parasite *Toxoplasma gondii*, underpinning its ability to replicate intracellularly and drive pathogenesis. Despite its importance, the molecular mechanisms governing this process remain incompletely understood. Here, we identify the PP2A-2 holoenzyme, potentially comprising a catalytic subunit (PP2A-C2), scaffolding subunit (PP2A-A2), and regulatory subunit (TgPR48, PP2A-B2), as a critical driver for daughter cell emergence. We further characterize two putative downstream effectors of PP2A-2, DCS1 and DCS2, demonstrating their essential roles in coordinating cytokinesis. Through a combination of conditional knockdown, functional complementation, and phosphoproteomic profiling, we uncover a tightly regulated network that orchestrates daughter cell formation. Disruption of PP2A-2 or its associated substrates results in significant defects in daughter cell separation and significantly impairs parasite proliferation. These findings reveal a previously unrecognized regulatory mechanism controlling *T. gondii* cell division and identify a promising molecular target for future therapeutic strategies.

## Introduction

Apicomplexan parasites constitute a diverse phylum of unicellular, obligate intracellular organisms that pose serious health threats to both humans and animals. Among them, *Plasmodium falciparum* is the causative agent of malaria [1], *Cryptosporidium* spp. are responsible for life-threatening diarrheal diseases [2], and *Toxoplasma gondii* is associated with encephalitis and congenital abnormalities [3,4]. Of these, *T. gondii* is the most widespread, capable of infecting virtually all warm-blooded animals and establishing chronic infections in nearly one-third of the global human population [3,4]. Although the parasite often remains dormant in immunocompetent hosts, its reactivation under immunosuppressive conditions triggers rapid expansion of the tachyzoite stage—leading to severe, and often fatal disease [5,6]. As tachyzoite replication directly drives pathogenesis, elucidating the molecular mechanisms controlling its growth and division is critical for identifying novel therapeutic targets.

*T. gondii* divides through a specialized process called endodyogeny, a unique form of internal budding in which two daughter cells develop within the mother cell′s cytoplasm [7–10]. This highly regulated mode of replication proceeds through a sequence of growth (G1), DNA synthesis (S), mitosis (M), and cytokinesis (C), with an extended G1 phase that ensures readiness for the subsequent cell cycle stages [7–10]. Central to this process is the parasite′s bipartite centrosome, composed of an inner core responsible for nuclear division and an outer core that coordinates daughter cell assembly [11,12]. Although historically considered to lack a G2 phase, recent findings suggest the presence of a G2-like interval that delays centrosome duplication and ensures mitotic fidelity [13].

A defining feature of apicomplexan division is internal budding, where daughter cells are constructed *de novo* within the mother cell. This process is orchestrated by the Inner Membrane Complex (IMC), a cytoskeletal scaffold beneath the plasma membrane essential for daughter cell formation [14–16]. Centrosome duplication initiates the recruitment of IMC proteins to the daughter cell scaffold (DCS), followed by the polymerization of subpellicular microtubules that drive cellular elongation [10,11]. Organelles are partitioned either through inheritance or *de novo* biogenesis, ensuring daughter cell viability. During late cytokinesis, the mother cell′s cytoskeleton disassembles, its plasma membrane remodels into new pellicles, and the remaining cytoplasmic material is packaged into a residual body [16].

To execute this intricate division program, *T. gondii* relies on complex signaling networks involving conserved kinases [7–10,17,18]. Key regulators include cyclin-dependent kinases (CDKs) [19–22], NIMA-related kinases [23], and Aurora kinases [24,25], which govern key cell cycle checkpoints. Additionally, *T. gondii* expresses apicomplexan-specific kinases, such as MAPKL1 and MAPK2, which control centrosome duplication and coordinate cell growth with division [26–29]. Despite growing knowledge of kinase-mediated regulation, the roles of protein phosphatases in parasite division remain largely unexplored [30].

Emerging evidence points to essential functions for phosphatases in apicomplexan cell cycle control. In *T. gondii*, phosphatases such as PP6 and PPKL have been linked to cytoskeletal organization and daughter cell development [31,32]. In mammalian cells, protein phosphatase 2A (PP2A) is a key regulator of cell cycle progression, influencing DNA replication timing, mitotic checkpoint control, and chromosomal segregation [33,34]. While *T. gondii* encodes two PP2A catalytic subunits (PP2A-C1 and PP2A-C2) [30], their specific roles are poorly characterized. Previous studies have shown that the PP2A-1 holoenzyme contributes to starch metabolism and bradyzoite differentiation [35]. Intriguingly, *T. gondii* expresses TgPR48, a homolog of the human PR48 regulatory subunit, which interacts with Cdc6 and modulates DNA replication in other systems [36]. TgPR48′s dynamic expression and localization during parasite division suggest a possible regulatory role in the tachyzoite cell cycle [35].

In this study, we identify and functionally characterize the PP2A-2 holoenzyme in *T. gondii*, likely composed of the catalytic subunit PP2A-C2 (TGME49_215170), the regulatory subunit TgPR48 (PP2A-B2, TGME49_200400), and the scaffolding subunit PP2A-A2 (TGME49_229350). Disruption of any component of this complex results in a marked defects in daughter cell emergence, implicating PP2A-2 as an essential regulator of late cytokinesis. Phosphoproteomic analysis revealed that two putative substrates, DCS1 and DCS2, become hyperphosphorylated upon PP2A-C2 depletion and are required for proper daughter cell emergence. Importantly, overexpression of TgPR48 partially rescued the cytokinesis defect in DCS2-depleted parasites, but failed to do so in those lacking DCS1, suggesting that these effectors operate in distinct or branching regulatory pathways. The study findings uncover a previously unrecognized role for PP2A-2–mediated dephosphorylation in orchestrating the final stages of *T. gondii* cell division. By elucidating the phosphatase-driven control of cytokinesis, this work opens new avenues for therapeutic strategies targeting parasite replication.

## Results

### The dynamic localization of TgPR48 is essential for parasite growth

**Fig 1:**
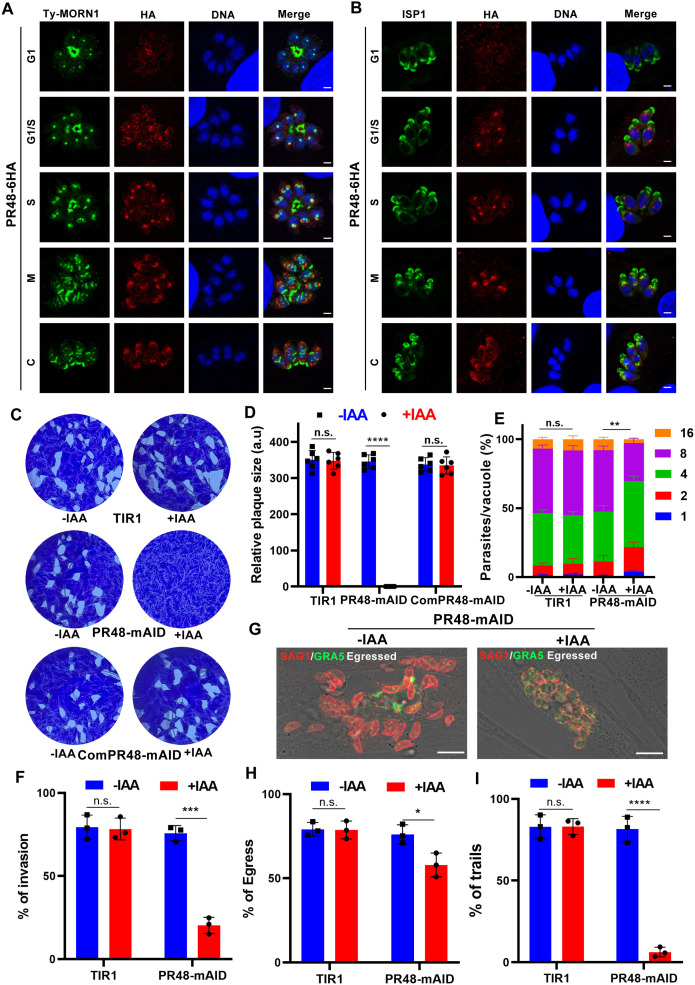
The TgPR48 subunit is essential for *Toxoplasma gondii* growth. (A, B). Immunofluorescence analysis of 6HA-tagged TgPR48 during the tachyzoite cell cycle. RH::PR48-6HA-infected cells were fixed 24 h post-infection and stained with anti-Ty antibody to label 2Ty-tagged MORN1 (A) or anti-ISP1 antibody (B) (green), anti-HA antibody to detect TgPR48 (red), and Hoechst dye to stain DNA (blue). Scale bar: 2 µm. (C). Representative images of plaque assays for the indicated strains cultured under standard conditions for 7 days, with or without 3-indoleacetic acid (IAA). Data are representative of six independent experiments. (D). Quantification of plaque formation by the indicated strains under standard conditions for 7 days with or without IAA. Data are presented as mean ± SD from six independent experiments. Statistical significance was assessed using a two-tailed, unpaired *t*-test. ****P < 0.0001; n.s., not significant. (E). Quantification of parasite replication in HFFs over 24 h with or without IAA (added 1 h post-invasion). Data are presented as mean ± SD from three independent experiments, analyzed by two-way ANOVA with Tukey’s multiple comparison test. **P = 0.0023. (F). Quantification of parasite invasion for the indicated strains grown on HFF monolayers with or without IAA. Data are presented as mean ± SD from three independent experiments, analyzed by a two-tailed, unpaired *t*-*t*est. ***P = 0.0001. (G). Representative images of egress assays for the indicated strains cultured under standard conditions with or without IAA for 36–40 h, followed by treatment with A23187 for 3 min. Parasites were labeled with anti-GRA5 (PVM marker, green) and anti-SAG1 (whole parasite marker, red). Images are representative of three independent experiments. Scale bar: 10 µm. (H). Quantification of egress assays. Data are presented as mean ± SD from three independent experiments, analyzed by a two-tailed, unpaired *t*-*t*est. *P = 0.0272. (I). Quantification of parasite motility for the indicated strains grown in the presence or absence of IAA and treated with A23187. Data are presented as mean ± SD from three independent experiments, analyzed by a two-tailed, unpaired *t*-*t*est. ****P < 0.0001.

Our previous study revealed that TgPR48 does not associate with the PP2A-1 core enzyme and exhibits a dynamic localization pattern throughout parasite division [35]. To further explore its subcellular localization, we performed co-staining with two well-characterized markers: MORN1, a centrocone marker, and ISP1, an apical cap marker. MORN1 is specifically localized to the ring-like structures at both the apical and basal ends of the inner membrane complex (IMC), as well as the spindle pole (centrocone), a structure crucial for organizing the mitotic spindle [37]. In contrast, ISP1 marks the apical cap region of the IMC and serves as an early indicator of daughter cell bud formation [38]. Our co-staining analysis revealed that TgPR48–6HA localizes to MORN1-defined rings near the centrocone during early S phase to early M phase. As mitosis progresses, TgPR48 translocates to the apical cap of the forming daughter cells, co-localizing with ISP1 from early M phase through C phase. In parasites at the G1 phase, TgPR48 predominantly localizes to the basal end, with this localization becoming more pronounced in larger parasitophorous vacuoles (PVs) containing more than eight tachyzoites (Fig 1A and 1B).

Given the significant negative fitness score (–3.17) observed in a genome-wide screen [39], we aimed to determine the biological role of TgPR48 using the mini auxin-inducible degron (mAID) system, a tool that has been successfully applied to study essential genes in *T. gondii* [40] (S1A Fig). For conditional knockdown, we generated the RH::PR48-mAID-6HA strain (referred to as PR48-mAID) by endogenously tagging TgPR48 with a C-terminal mAID-6HA in an RHΔ*ku80*Δ*hxgprt*::TIR1–3Flag background, which expresses the auxin receptor TIR1. The mAID-tagged TgPR48 retained the dynamic localization pattern observed with the 6HA-tagged version (S1B Fig). Addition of 3-indoleacetic acid (IAA or auxin) resulted in rapid degradation of TgPR48, confirmed by both immunofluorescence microscopy (S1B Fig) and immunoblotting (S1C Fig).

To assess the impact of TgPR48 depletion on parasite growth, we performed plaque assays to evaluate the lytic cycle of PR48-mAID parasites grown in the presence or absence of IAA for 7 days. While the parental strain formed normal plaques regardless of IAA treatment, PR48-mAID parasites failed to form plaques following TgPR48 depletion (Fig 1C and 1D). This result demonstrates that TgPR48 is essential for the progression of the lytic cycle in *T. gondii*. Subsequent analyses revealed that intracellular replication efficiency (Fig 1E) and host cell invasion (Fig 1F) were significantly reduced in TgPR48-depleted parasites. Although these parasites retained the ability to egress from the PV, evidenced by disruption of the PV membrane (PVM) as indicated by GRA5 staining, gliding motility was severely impaired (Fig 1G-I). These motility defects led to parasites clustering around lysed PVs. While egress still occurred, the egressed parasites exhibited abnormal clustering and a rounded morphology, instead of the typical crescent shape (Fig 1G). Moreover, a microneme secretion assay, triggered by ethanol, demonstrated that TgPR48 depletion impaired MIC2 secretion, which likely contributes to the observed defects in invasion and motility (S1D Fig).

To confirm that these phenotypic defects were specifically due to the loss of TgPR48, we complemented the PR48-mAID strain by introducing a non-degradable, wild-type copy of TgPR48 under its endogenous promoter into the *uprt* locus. This complementation fully rescued the defects observed in TgPR48-depleted parasites, as evidenced by restored plaque formation (Fig 1C and 1D) and normal immunofluorescence localization (S1B Fig). These results conclusively demonstrate that TgPR48 is essential for parasite replication, invasion, and motility, emphasizing its critical role in parasite growth and survival.

### TgPR48 is essential for daughter cell emergence and parasite morphology

**Fig 2:**
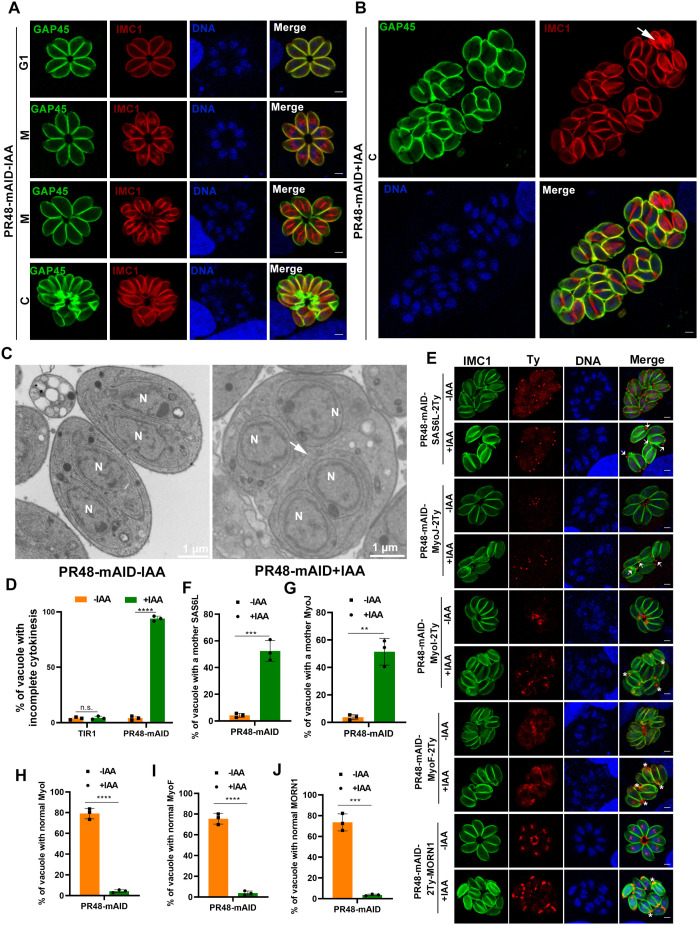
The TgPR48 subunit is essential for daughter cell emergence. (A). Immunofluorescence images showing successful daughter cell emergence in TgPR48-expressing parasites. Cells infected with TgPR48-expressing parasites were fixed 24 h post-infection without IAA treatment and stained with anti-GAP45 (green, mother cell) and anti-IMC1 (red, daughter cell). Images are representative of three independent biological replicates. Scale bar: 2 µm. (B). Immunofluorescence images showing defective daughter cell emergence in TgPR48-depleted parasites. RH::PR48-mAID parasites were fixed 32 h post-infection with IAA treatment and stained with anti-GAP45 (green) and anti-IMC1 (red). Arrows indicate tethered daughter cells undergoing a new division cycle. Images are representative of three independent biological replicates. Scale bar: 2 µm. (C). Representative electron microscopy images of intracellular RH::PR48-mAID parasites treated for 24 h with or without IAA. Arrows indicate the inner membrane complex (IMC) of daughter cells facing each other in the absence of a plasma membrane. N, cell nucleus. Scale bar: 1 µm. (D). Quantification of vacuoles containing defective daughter cell emergence, defined as two or more mature daughter parasites within the same plasma membrane. Data are presented as mean ± SD from three independent experiments. Statistical significance was assessed using a two-tailed, unpaired *t*-test. ****P < 0.0001; n.s., not significant. (E). Immunofluorescence images showing the impact of TgPR48 depletion on late cytokinesis. RH::PR48-mAID-infected cells were fixed 24–28 h post-infection with IAA treatment and stained with antibodies against SAS6L-2Ty, MyoJ-2Ty, MyoI-2Ty, MyoF-2Ty, and 2Ty-MORN1 to visualize respective markers. Arrows indicate remnant mother conoids or basal complexes, and asterisks indicate disorganized parasites. Images are representative of three independent experiments. Scale bar: 2 µm. (F-J). Quantification of vacuoles with a remnant mother conoid (SAS6L) ***P = 0.0005 (F), basal complex (MyoJ) **P = 0.0011 (G), and organized parasites (MyoI) ****P < 0.0001 (H), MyoF ****P < 0.0001 (I), and MORN1 ***P = 0.0001 (J) for RH::PR48-mAID parasites grown with or without IAA. Data are presented as mean ± SD from three independent experiments. Statistical significance was assessed using a two-tailed, unpaired *t*-test.

Given its dynamic localization on the daughter cell scaffold, TgPR48 is likely crucial for both daughter cell development and parasite morphology. To investigate this, we treated PR48-mAID parasites with IAA for 32 h and stained them with IMC1, a marker for developing daughter cell scaffolds and the mother cell pellicle. To further examine pellicle maturation, we used GAP45, a core glideosome component involved in the attachment of the plasma membrane (PM) to the IMC. GAP45 is initially associated with the parental pellicle and later recruited to the daughter pellicle during cytokinesis [41]. In untreated parasites, GAP45 was detected exclusively on the mother cell pellicle, with no signal on newly formed daughter scaffolds marked by IMC1. However, as daughter cells matured and emerged, GAP45 co-localized with IMC1, indicating proper pellicle maturation (Fig 2A).

After 32 h of TgPR48 depletion, GAP45 localization was significantly disrupted. Mature daughter cells exhibited only partial GAP45 coverage, with GAP45 predominantly restricted to the outer pellicle of daughter cells tethered together, initiating a new division cycle (Fig 2B). Additional glideosome markers, such as MLC1 (which defines regions of the IMC) and SAG1 (a PM marker), displayed irregular staining patterns in newly formed daughter cells of TgPR48-depleted parasites (S1E Fig). Moreover, after 12 h of IAA treatment, incomplete pellicle assembly was observed (S1F Fig). As replication progressed, only a small proportion of parasites were able to rupture the host cell and egress; however, their subsequent invasion capacity was severely impaired.

To further investigate the structural defects resulting from TgPR48 depletion, we used transmission electron microscopy (TEM), which revealed severe morphological abnormalities, including incomplete plasma membrane formation in mature daughter cells (Fig 2C). Quantitative analysis confirmed that the majority of TgPR48-depleted parasites failed to complete cytokinesis, preventing proper daughter cell emergence from the mother cell (Fig 2D). Given that the PP2A-1 holoenzyme is involved in bradyzoite differentiation and starch metabolism, we assessed whether TgPR48 participates in this process. We generated a PR48-mAID strain in the Pru::TIR1 background using a strategy similar to that employed in the RH::TIR1 strain (S1G Fig). Our results showed that TgPR48 depletion did not affect bradyzoite differentiation, confirming that TgPR48 is not involved in the PP2A-1 holoenzyme pathway (S1H and S1I Fig).

### TgPR48 depletion selectively affects cytokinesis without disrupting organelle biogenesis

To evaluate whether TgPR48 depletion influences early cell cycle progression or organelle formation, we performed immunofluorescence assays (IFA) using established markers for key cellular structures. These included centrosome markers (SAS6 and Sfi1 for the outer core, CEP250-L1 for the inner core), kinetochore (Nuf2), centromere (Chromo1), micronemes (MIC2), apicoplast (CPN60), dense granules (GRA5), rhoptries (ARO), and mitochondria (HSP60). In TgPR48-depleted parasites, these structures appeared largely unaffected during early cell division stages, suggesting that early events such as organelle formation and the initial phases of cell cycle progression were intact (S2 Fig). Interestingly, mitochondria in TgPR48-depleted parasites occasionally appeared collapsed, irregular, and mislocalized outside the daughter cells. These abnormalities may arise from aberrant parasite morphology and incomplete cytokinesis, reflecting secondary effects from disrupted cytoskeletal remodeling and failed cytokinesis.

In contrast to early-stage structures, analysis using late-stage markers, including SAS6L (conoid), RNG1 and RNG2 (apical polar ring), MyoI (residual body), MyoJ (basal complex constriction), MyoF (actin organization), and MORN1 (basal complex scaffolding), revealed significant structural disorganization. The characteristic rosette arrangement of tachyzoites was disrupted following TgPR48 depletion (Figs 2E to 2J, and S2). Although conoid and apical/basal complexes in mature daughter cells were detectable, remnants of the mother conoid, apical, and basal complexes still persisted in a significant proportion of vacuoles from TgPR48-depleted parasites (Fig 2E to 2J), indicating incomplete remodeling during the final stages of cytokinesis.

### PP2A-2 catalytic subunit may interact with TgPR48 to drive daughter cell emergence

Given the dynamic localization of TgPR48 during parasite division, we sought to identify its functional partners using the TurboID proximity biotin labeling method. This technique enabled the detection of interacting or neighboring proteins, including potential substrates. We generated the PR48-TurboID-4Ty strain by fusing the TurboID-4Ty fragment to the C-terminus of the endogenous TgPR48 gene. IFA confirmed that the PR48-TurboID-4Ty fusion retained the same localization pattern as TgPR48–6HA (S3A Fig), indicating that the fusion did not disrupt TgPR48’s dynamic localization. To assess TurboID activity, we treated PR48-TurboID-4Ty parasites with biotin and detected biotinylated proteins via streptavidin staining in both IFA and Western blotting. IFA revealed a strong fluorescent signal near the TgPR48 localization site, distinct from the biotinylated apicoplasts (S3A Fig). Western blotting further confirmed a dramatic increase in biotinylated proteins compared to untreated controls (S3B Fig), validating the strong labeling activity of PR48-TurboID-4Ty.

To identify potential TgPR48-interacting proteins, we performed mass spectrometry on biotinylated proteins isolated from PR48-TurboID-4Ty and parental strain parasites treated with D-biotin. By filtering for proteins that were enriched at least two-fold and displayed a spectral count greater than three, we identified 400 potential TgPR48-interacting or neighboring proteins. Interestingly, this list included the PP2A-2 catalytic subunit (TGME49_215170) (S1 Table).

**Fig 3:**
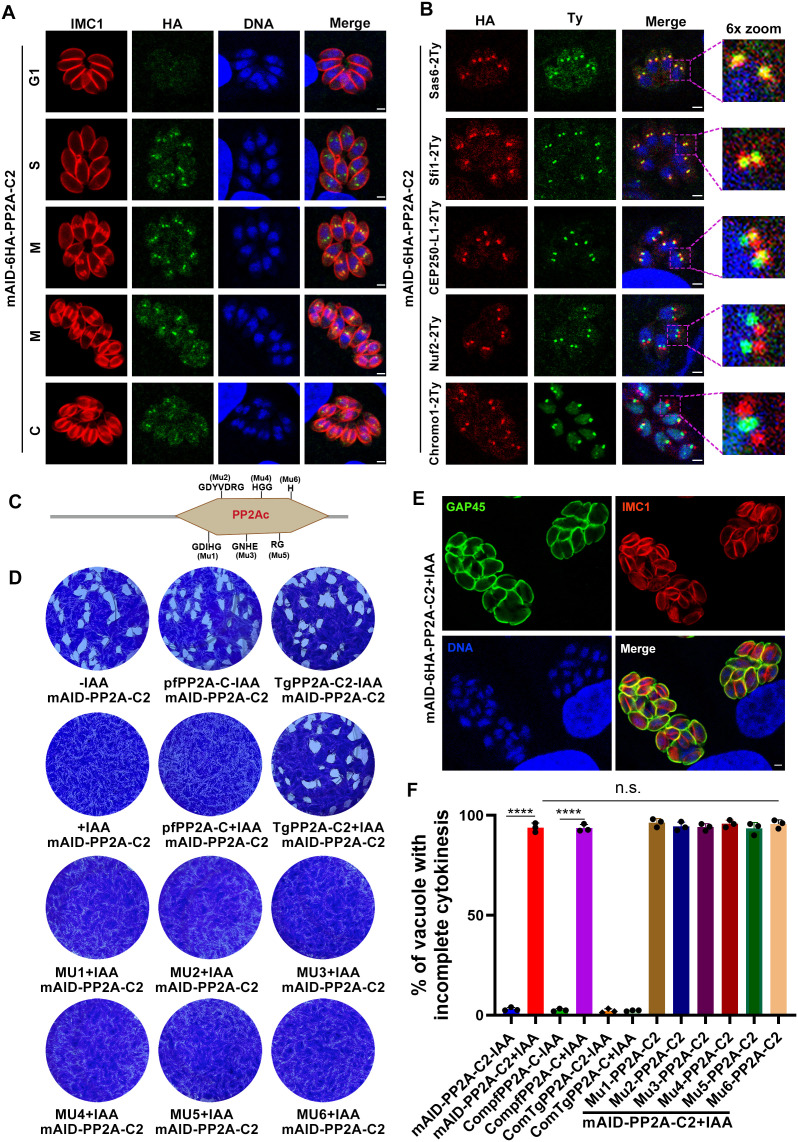
Depletion of PP2A-C2 mimics the phenotype observed in TgPR48-depleted parasites. (A). Immunofluorescence analysis of mAID-6HA-tagged PP2A-C2 during the tachyzoite cell cycle. RH::mAID-6HA-PP2A-C2-infected cells were fixed 24 h post-infection and stained with anti-IMC1 antibody (red) to visualize the cell cycle, anti-HA antibody to detect PP2A-C2 (green), and Hoechst dye to stain DNA (blue). Scale bar: 2 µm. (B). Confocal analysis of PP2A-C2 co-localization with various cellular markers, including the outer centrosome core markers SAS6-2Ty and Sfi1-2Ty, the inner centrosome core marker CEP250-L1-2Ty, the kinetochore marker Nuf2-2Ty, and the centromere marker Chromo1-2Ty, reveals that PP2A-C2 localizes to the outer centrosome core. RH::mAID-6HA-PP2A-C2-infected cells were fixed 24 h post-infection and stained with anti-Ty antibody (green) to visualize the indicated marker, anti-HA antibody to detect PP2A-C2 (red), and Hoechst dye to stain DNA (blue). Scale bar: 2 µm. (C). Schematic representation of the PP2A-C2 gene model highlighting the six consensus sequences of the core catalytic motif. (D). Representative images of plaque assays of the indicated strains cultured under standard conditions for 7 days, with or without IAA. Images are representative of six independent experiments. (E). Representative immunofluorescence images showing defective daughter cell emergence in PP2A-C2-depleted parasites. Cells infected with RH::mAID-6HA-PP2A-C2 parasites were fixed 32 h post-infection with IAA treatment and stained with anti-GAP45 antibody (green) and anti-IMC1 antibody (red). Images are representative of three independent biological replicates. Scale bar: 2 µm. (F). Quantification of vacuoles containing parasites with defective daughter cell emergence. Vacuoles with two or more mature daughter parasites enclosed within the same plasma membrane were quantified. Data are presented as mean ± SD from three independent experiments. Statistical significance was determined using a two-tailed, unpaired *t*-test. ****P < 0.0001; n.s., not significant.

Given that TgPR48 shares homology with human PP2A regulatory subunits, we focused our investigation on the PP2A-2 catalytic subunit. While attempts to tag the C-terminus of PP2A-C2 with mAID-6HA were unsuccessful, likely due to the functional importance of the C-terminal region or its susceptibility to post-translational cleavage or degradation, we successfully tagged PP2A-C2 at the N-terminus with mAID-6HA (S3C Fig). Interestingly, IFA revealed that PP2A-C2 exhibits dynamic localization throughout the parasite cell cycle. During early S and M phases, PP2A-C2 accumulated at the centrosome, whereas in the C and G1 phases, it became nearly undetectable (Fig 3A). Co-localization experiments with centrosomal markers, including SAS6–2Ty and Sfi1–2Ty (outer centrosome core) [11], CEP250-L1-2Ty (inner centrosome core) [11], Nuf2–2Ty (kinetochore) [42], and Chromo1–2Ty (centromere) [43], confirmed that PP2A-C2 specifically localizes to the outer centrosome core (Fig 3B).

To evaluate the functional role of PP2A-C2, we depleted this protein using the mAID system. Following 24 h of treatment with IAA, immunofluorescence and immunoblotting analyses confirmed efficient protein degradation (S3D and S3E Fig). Remarkably, PP2A-C2 depletion resulted in defects similar to those observed upon TgPR48 depletion, including impaired daughter cell emergence and a complete loss of plaque formation after 7 days of growth (Fig 3C to 3F). Previous research has suggested that PP2A-C2 may be a substrate of the CDK-related kinase CRK4, with its localization or function potentially depending on CRK4 activity [13]. However, our results showed that depletion of CRK4 did not alter the localization of PP2A-C2, which remained enriched at the centrosome (S3F Fig).

### Catalytic activity of PP2A-C2 is essential for parasite viability

As a member of the PPP family, PP2A catalytic subunit is highly conserved across eukaryotes and contains six core motifs (GDIHG, GDYVDRG, GNHE, HGG, RG, and H) involved in metal ion chelation (Fig 3C) [30,33]. To investigate whether the catalytic activity of PP2A-C2 is essential for parasite viability, we generated wild-type and mutant forms of these core motifs. The constructs, each containing an N-terminal 2Ty tag and driven by the native promoter, were inserted into the *uprt* locus of the mAID-6HA-PP2A-C2 background strain. While complementation with wild-type PP2A-C2 fully rescued the defects observed upon PP2A-C2 depletion, catalytically inactive mutants failed to restore daughter cell emergence (Figs 3D, 3F, and S3G). Interestingly, mutations in the conserved core motifs of PP2A-C2, excluding the RG (Mu5) and H (Mu6) motifs, disrupted its subcellular localization, preventing its accumulation at the outer centrosomal core (S3G Fig). These mutations also resulted in reduced PP2A-C2 protein levels (S3H Fig), suggesting that the integrity of these catalytic motifs is critical not only for proper localization but also for protein stability and expression.

To explore whether PP2A-C2 function is conserved across species, we expressed the homologous PP2A-C2 protein from *Plasmodium falciparum* (PF3D7_0925400) in *T. gondii*. Sequence alignment using BLAST in VEuPathDB (https://veupathdb.org) revealed a highly significant E-value of 1e − 138, with 56% identity (188/335 amino acids), 70% positives (233/335), and 8% gaps (28/335). The PfPP2A-C protein was successfully expressed in *T. gondii* (S3H Fig), driven by the TgPP2A-C2 promoter, and localized to both the nucleus and cytoplasm, with predominant accumulation in the nucleus (S3G Fig). However, this heterologous complementation failed to rescue the phenotype of PP2A-C2-depleted parasites (Figs 3D and 3F, and S3G), suggesting substantial functional divergence between PP2A-C2 orthologs in these species. This divergence may reflect species-specific regulatory mechanisms, differences in substrate interactions, or distinct holoenzyme compositions.

### PP2A-C2 forms a stable core complex with PP2A-A2 and potentially associates with TgPR48

**Fig 4:**
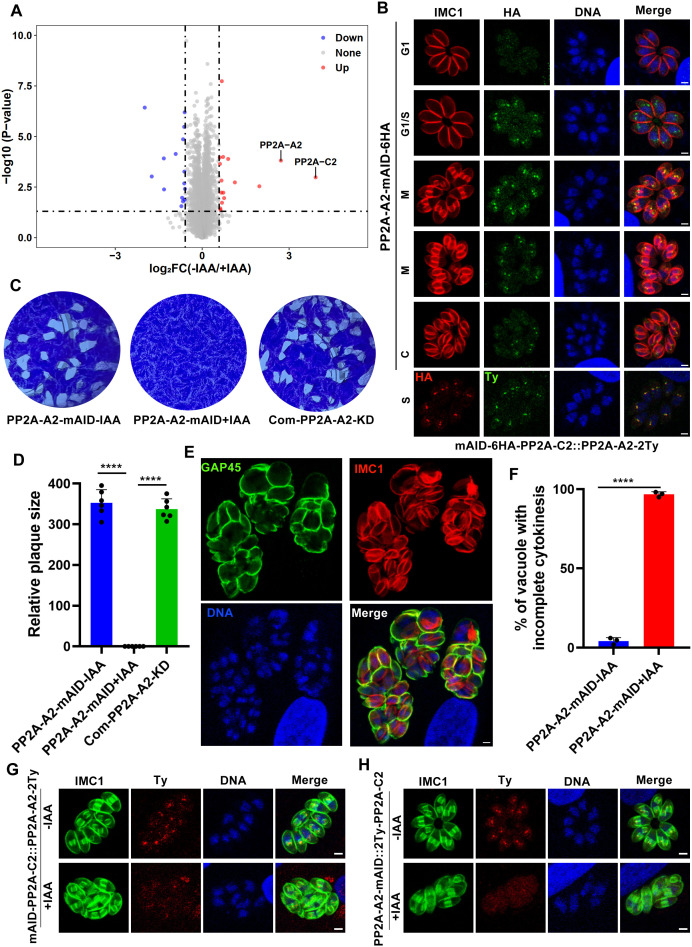
Proteomic analysis identifies PP2A-A2 as the potential structural subunit of the PP2A-2 holoenzyme. (A). Volcano plot showing differentially expressed proteins in PP2A-C2-expressing and PP2A-C2-depleted strains cultured under normal conditions for 2 days. Data from four biological replicates were analyzed, with a fold change of ≥ 1.5 or ≤ −1.5 and a *P* value < 0.05 considered statistically significant. (B). Immunofluorescence analysis of mAID-6HA-tagged PP2A-A2 or RH::mAID-6HA-PP2A-C2::PP2A-A2-2Ty during the tachyzoite cell cycle. RH::PP2A-A2-mAID-6HA or RH::mAID-6HA-PP2A-C2::PP2A-A2-2Ty-infected cells were fixed 24 h post-infection and stained with anti-IMC1 antibody (red) or anti-HA to visualize the cell cycle or PP2A-C2, anti-HA or anti-Ty antibody (green) to detect PP2A-A2, and Hoechst dye (blue) to stain DNA. Scale bar: 2 µm. (C). Representative images of plaque assays of the indicated strains cultured under standard conditions for 7 days with or without IAA. Images are representative of six independent experiments. (D). Quantification of plaques formed by the indicated strains cultured for 7 days under standard conditions with or without IAA. Data represent the mean ± SD from six independent experiments. Statistical significance was determined using a two-tailed, unpaired *t*-*t*est. ****P < 0.0001; n.s., not significant. (E). Representative immunofluorescence images showing defective daughter cell emergence in PP2A-A2-depleted parasites. RH::PP2A-A2-mAID-6HA-infected cells were fixed 32 h post-infection with IAA treatment and stained with anti-GAP45 antibody (green) and anti-IMC1 antibody (red). Images are representative of three independent biological replicates. Scale bar: 2 µm. (F). Quantification of vacuoles containing parasites with defective daughter cell emergence. Vacuoles with two or more mature daughter parasites enclosed within the same plasma membrane were quantified. Data represent the mean ± SD from three independent experiments. Statistical significance was determined using a two-tailed, unpaired *t*-tes*t*. ****P < 0.0001; n.s., not significant. (G). Depletion of PP2A-C2 affects the localization of PP2A-A2. RH::mAID-6HA-PP2A-C2::PP2A-A2-2Ty-infected cells treated with IAA were fixed 24 h post-infection and stained with anti-IMC1 antibody (green) to visualize the parasites, anti-Ty antibody (red) to detect PP2A-A2, and Hoechst dye (blue) to stain DNA. Images are representative of three independent biological replicates. Scale bar: 2 µm. (H). Depletion of PP2A-A2 affects the localization of PP2A-C2. RH::PP2A-A2-mAID-6HA::2Ty-PP2A-C2-infected cells treated with IAA were fixed 24 h post-infection and stained with anti-IMC1 antibody (green) to visualize the parasites, anti-Ty antibody (red) to detect PP2A-C2, and Hoechst dye (blue) to stain DNA. Images are representative of three independent biological replicates. Scale bar: 2 µm.

Given the catalytic role of PP2A-C2, we performed quantitative proteomic and phosphoproteomic analyses on the mAID-6HA-PP2A-C2 strain, with and without IAA treatment, to investigate the molecular mechanisms underlying the defects in daughter cell emergence observed upon PP2A-C2 depletion. Using the advanced 10X Proteomics platform, which enhances proteomic analysis, we identified 5,518 comparable proteins across four biological replicates (S2 Table). The quantitative proteomics revealed 31 proteins with significant abundance changes (≥ 1.5-fold, **P* *< 0.05) following PP2A-C2 depletion, with 16 proteins downregulated and 15 upregulated (S3 Table). As expected, PP2A-C2 showed the largest reduction in abundance compared to the control strain. Interestingly, TGME49_229350 (referred to as PP2A-A2) was the second most downregulated protein, suggesting a functional link between PP2A-C2 and PP2A-A2 (Fig 4A).

Structural predictions indicated that TGME49_229350 contains 15 tandem HEAT (Huntingtin-Elongation-A subunit-TOR) repeats [33,44,45], forming an elongated horseshoe-shaped structure similar to PP2A scaffolding subunits (S4A Fig). This structural similarity led us to hypothesize that TGME49_229350 acts as the PP2A-A2 scaffolding subunit. To test this, we endogenously tagged PP2A-A2 with mAID-6HA at its C-terminus and examined its localization and function. Similar to PP2A-C2, PP2A-A2 exhibited dynamic centrosomal localization during early cell division and co-localized with PP2A-C2 (Fig 4B). Following IAA treatment, immunofluorescence and immunoblotting confirmed the successful degradation of PP2A-A2 (S4B, S4C Fig). Interestingly, parasites depleted of PP2A-A2 exhibited severe morphological abnormalities and failed to form plaques after 7 days of growth, phenotypes identical to those observed upon PP2A-C2 depletion (Fig 4C-F). Interestingly, PP2A-A2 was identified as a putative TgPR48 interactor in the TurboID assay. Structural predictions using AlphaFold 3 suggested that TgPR48, PP2A-C2, and PP2A-A2 may form a complex (S4D Fig) [44,45]. Based on these findings, we designated TGME49_229350 as the PP2A-A2 scaffolding subunit and TgPR48 as the PP2A-B2 regulatory subunit.

To further elucidate the relationships within this PP2A-2 complex, we performed a series of endogenous tagging and depletion experiments. These included tagging PP2A-A2-2Ty in the mAID-PP2A-C2 strain, PP2A-A2-2Ty in the PR48-mAID strain, 2Ty-PP2A-C2 in the PP2A-A2-mAID strain, 2Ty-PP2A-C2 in the PR48-mAID strain, TgPR48–2Ty in the PP2A-A2-mAID strain, and TgPR48–2Ty in the mAID-PP2A-C2 strain. IFA and immunoblotting analyses revealed that depletion of PP2A-C2 disrupted both the localization and protein levels of PP2A-A2. Conversely, depletion of PP2A-A2 similarly affected the localization and protein levels of PP2A-C2 (Figs 4G and 4H; S4E and S4F). However, depletion of either PP2A-C2 or PP2A-A2 did not impact TgPR48 localization. Similarly, TgPR48 depletion had no effect on PP2A-C2 or PP2A-A2 localization, despite TgPR48 co-localizing with both PP2A-C2 and PP2A-A2 during the S phase (S4G Fig).

These findings suggest that PP2A-C2 and PP2A-A2 form a stable dimeric core complex, while TgPR48 transiently associates with this complex specifically during the S phase. This transient interaction is further supported by co-localization patterns observed between TgPR48 and PP2A-C2 (S4H Fig). Collectively, the results suggest that PP2A-C2 operates within a stable core complex with PP2A-A2, while TgPR48 serves as a regulatory subunit that associates with the complex in a cell cycle-dependent manner. This complex plays a critical role in daughter cell emergence and underscores its importance for parasite viability and cell division. Further studies to confirm this complex may provide valuable insights into parasite biology and potential therapeutic targets.

### Phosphoproteomic analysis identifies putative PP2A-2 holoenzyme substrates

**Fig 5:**
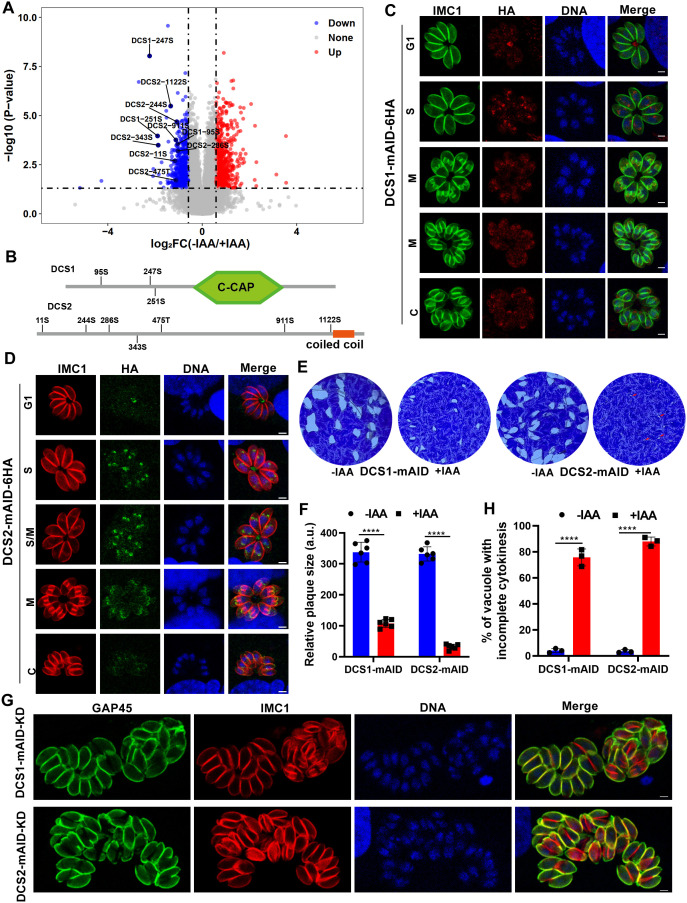
Comparative phosphoproteomic analysis identifies potential substrates of the PP2A-2 holoenzyme. (A). Volcano plot showing differentially phosphorylated peptides in PP2A-C2-expressing and PP2A-C2-depleted strains cultured under normal conditions for 2 days. Data from four biological replicates were analyzed, with a fold change of ≥ 1.5 or ≤ −1.5 and a *P* value < 0.05 considered statistically significant. (B). Schematic representation of the DCS1 and DCS2 gene models highlighting key domains and hyperphosphorylated sites identified following PP2A-C2 depletion. (C). Immunofluorescence analysis of mAID-6HA-tagged DCS1 during the tachyzoite cell cycle. RH::DCS1-mAID-6HA-infected cells were fixed 24 h post-infection and stained with anti-IMC1 antibody (green) to visualize the cell cycle, anti-HA antibody (red) to detect DCS1, and Hoechst dye (blue) to stain DNA. Scale bar: 2 µm. (D). Immunofluorescence analysis of mAID-6HA-tagged DCS2 during the tachyzoite cell cycle. RH::DCS2-mAID-6HA-infected cells were fixed 24 h post-infection and stained with anti-IMC1 antibody (red) to visualize the cell cycle, anti-HA antibody (green) to detect DCS2, and Hoechst dye (blue) to stain DNA. Scale bar: 2 µm. (E). Representative images of plaque assays for the indicated strains cultured under standard conditions for 7 days with or without IAA. Images are representative of six independent experiments. (F). Quantification of plaques formed by the indicated strains cultured for 7 days under standard conditions with or without IAA. Data represent the mean ± SD from six independent experiments. Statistical significance was determined using a two-tailed, unpaired *t*-*t*est. ****P < 0.0001. (G). Representative immunofluorescence images showing defective daughter cell emergence in DCS1- or DCS2-depleted parasites. RH::DCS1-mAID-6HA or RH::DCS2-mAID-6HA-infected cells were fixed 32 h post-infection with IAA treatment and stained with anti-GAP45 antibody (green) and anti-IMC1 antibody (red). Images are representative of three independent biological replicates. Scale bar: 2 µm. (H). Quantification of vacuoles containing parasites with defective daughter cell emergence. Vacuoles with two or more mature daughter parasites enclosed within the same plasma membrane were quantified. Data represent the mean ± SD from three independent experiments. Statistical significance was determined using a two-tailed, unpaired *t*-tes*t*. ****P < 0.0001.

Our phosphoproteomic analysis identified 23,230 phosphosites across 3,414 proteins, with phosphoserine modifications being the most prevalent (S4 Table). Quantitative data were obtained for 19,548 of these sites, spanning 3,288 proteins, which allowed for normalization to account for differences in protein abundance between experimental conditions. This normalization ensured that the observed changes in phosphorylation reflected actual modifications rather than variations in protein expression. After normalization, we identified 1,257 phosphopeptides exhibiting significant changes in phosphorylation levels (fold change ≥ 1.5 or ≤ −1.5) between PP2A-C2-expressed and -depleted strains. Of these, 616 phosphosites on 495 proteins showed increased phosphorylation upon PP2A-C2 depletion, while 641 phosphosites on 505 proteins exhibited reduced phosphorylation levels (Fig 5A and S5 Table).

The PP2A regulatory subunit plays a crucial role in directing the PP2A holoenzyme’s activity by modulating its temporal and spatial specificity [33]. Given the dynamic localization of TgPR48, we sought to identify hyperphosphorylated proteins exhibiting similar expression patterns, which may serve as potential substrates of the PP2A-2 holoenzyme. Since the HyperLOPIT data on daughter cell scaffold proteins is limited [46], we leveraged the Toxo Single Cell Atlas (https://umbibio.math.umb.edu/toxosc/), which provides scRNA-seq data on rapidly dividing *T. gondii* tachyzoites [47]. We cross-referenced proteins with expression profiles resembling TgPR48 with the PR48-TurboID interaction data, selecting four candidates for further investigation: TGME49_216240, TGME49_231790, TGME49_260580, and TGME49_236560 (S5A, S5B Fig). While this study was in preparation, an independent study identified TGME49_216240 as Daughter Cell Scaffold protein 1 (DCS1) and TGME49_231790 as Daughter Cell Scaffold protein 2 (DCS2) [48]. To maintain consistency and clarity, we have adopted these designations in our study.

### DCS1 and DCS2 depletion severely disrupts daughter cell emergence

Structurally, DCS1 contains a C-CAP/Cofactor C-like domain, a motif typically found in cytoskeleton-associated proteins that aids in α- and β-tubulin heterodimer assembly (Fig 5B). In contrast, DCS2, TGME49_260580, and TGME49_236560 lack identifiable functional domains, except for predicted coiled-coil regions. Given the absence of HyperLOPIT localization data for DCS1, DCS2, and TGME49_260580, while TGME49_236560 is predicted to localize in the nucleus [46], we endogenously tagged their C-termini with mAID-6HA to assess their localization and function. As expected, the expression and localization of DCS1 and DCS2 closely mirrored TgPR48 (Fig 5C, 5D). Upon IAA treatment, both proteins were rapidly degraded, as confirmed by immunoblotting (S5C, S5D Fig). Interestingly, TGME49_260580 and TGME49_236560 also exhibited cell cycle-dependent expression (S5E, S5F Fig). However, their depletion did not impact daughter cell emergence (S5G, S5H Fig), leading us to exclude them from further analysis.

Phenotypic analysis revealed that DCS2 depletion resulted in defects resembling those seen with TgPR48 depletion, causing severe growth defects and almost complete absence of plaque formation. In contrast, DCS1 depletion led to smaller plaques, indicating a milder phenotype (Fig 5E, 5F). Further quantitative assessments showed that DCS2-depleted parasites exhibited significantly more pronounced defects in parasite growth and daughter cell emergence compared to DCS1-depleted strains (Fig 5G, 5H). Our phosphoproteomic analysis highlights the extensive impact of PP2A-C2 depletion on phosphorylation dynamics. Through integrative expression and interaction profiling, we identified DCS1 and DCS2 as potential downstream effectors regulated by the PP2A-2 holoenzyme, with DCS2 playing a particularly critical role in daughter cell emergence.

### Phosphorylation of DCS1 and DCS2 and their relationship with the PP2A-2 holoenzyme

**Fig 6:**
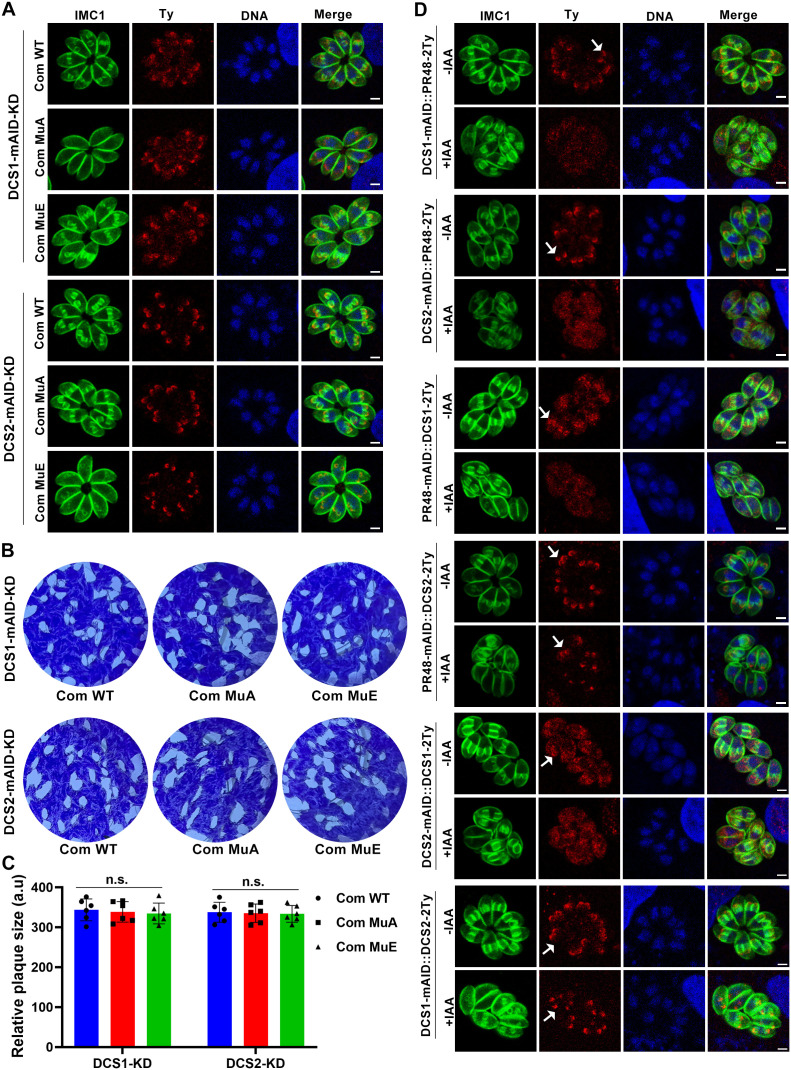
The identified phosphorylation sites are not essential for the localization or function of DCS1 or DCS2. (A). Immunofluorescence analysis of phosphomutant DCS1 and DCS2 strains shows that the identified phosphorylation sites are not essential for their activity. The indicated strains were treated with IAA for 24 h to induce the depletion of DCS1 or DCS2. Parasites were visualized using IMC1, and Ty-tagged complemented proteins were detected using an anti-Ty antibody. Scale bar: 2 µm. (B). Representative images of plaque assays of the indicated strains cultured under standard conditions for 7 days with IAA treatment. Images are representative of six independent experiments. (C). Quantification of plaques formed by the indicated strains cultured under standard conditions for 7 days with IAA treatment. Data are presented as mean ± SD from six independent experiments. Statistical significance was assessed using a two-tailed, unpaired *t*-test. n.s., not significant. (D). Immunofluorescence analysis of the localization of TgPR48, DCS1, and DCS2 following depletion of mAID-tagged proteins. The indicated strains were treated with IAA for 24 h to induce protein depletion. Ty-tagged proteins were detected using an anti-Ty antibody, while IMC1 staining was used to visualize the parasites. The arrow indicates the typical localization of the Ty-tagged protein. Scale bar: 2 µm.

Phosphoproteomic analysis revealed that multiple serine and threonine residues in DCS1 (95S, 247S and 251S) and DCS2 (11S, 244S, 286S, 343S, 475T, 911S, and 1122S) were hyperphosphorylated in the PP2A-C2-depleted strain, suggesting that these proteins may serve as potential substrates of the PP2A-2 holoenzyme. To explore this further, we generated phosphomimetic (E) and phosphorylation-defective (A) mutants by substituting serine or threonine residues with glutamic acid (E) to mimic constitutive phosphorylation, or with alanine (A) to simulate a non-phosphorylatable state. Unexpectedly, these mutations did not affect the function or localization of DCS1 or DCS2. Both the phosphomimetic (E) and phosphorylation-defective (A) mutants were able to rescue the phenotypic defects observed upon DCS1 or DCS2 depletion. Additionally, the mutant DCS proteins retained their correct localization within the tachyzoites (Fig 6A–6C). This indicates that the phosphorylation status of these proteins may not be the sole determinant of their function or activity, suggesting that other regulatory mechanisms may also play a critical role.

### Interplay between PP2A-2 holoenzyme, DCS1, and DCS2

To investigate the relationship between the PP2A-2 holoenzyme, DCS1, and DCS2, we examined their localization dynamics following targeted degradation of each component. We found that depletion of PP2A-C2 and PP2A-A2 did not affect the localization of TgPR48, DCS1, or DCS2. This suggests that centrosome-localized PP2A-C2 and PP2A-A2 are not required for the proper positioning of TgPR48, DCS1, or DCS2 at the apical pole of daughter cells (S6 Fig). Conversely, degradation of TgPR48, DCS1, or DCS2 did not disrupt the localization of PP2A-C2 or PP2A-A2 at the centrosome (S6 Fig). However, a clear interdependence between the localization of TgPR48, DCS1, and DCS2 was observed. TgPR48 depletion led to a loss of DCS1 enrichment at the apical pole of daughter cells, causing it to disperse throughout the cytoplasm. In turn, DCS1 depletion caused TgPR48 to become more diffuse in the cytoplasm, rather than remaining concentrated at its typical localization site. These reciprocal localization dependencies suggest a crucial role for DCS1 in maintaining TgPR48’s apical targeting (Fig 6D). In contrast, TgPR48 depletion did not affect DCS2 localization, which remained apically enriched. However, depletion of DCS2 caused TgPR48 to mislocalize from its concentrated apical region to a more diffuse cytoplasmic pattern (Fig 6D). Importantly, DCS1 depletion did not alter DCS2 localization, but DCS2 depletion led to a shift in DCS1 positioning (Fig 6D). These findings highlight the essential roles of DCS1 and DCS2 in the proper localization of TgPR48 at the apical pole of daughter cells.

### TgPR48 overexpression partially rescues DCS2, but not DCS1, depletion defects

**Fig 7:**
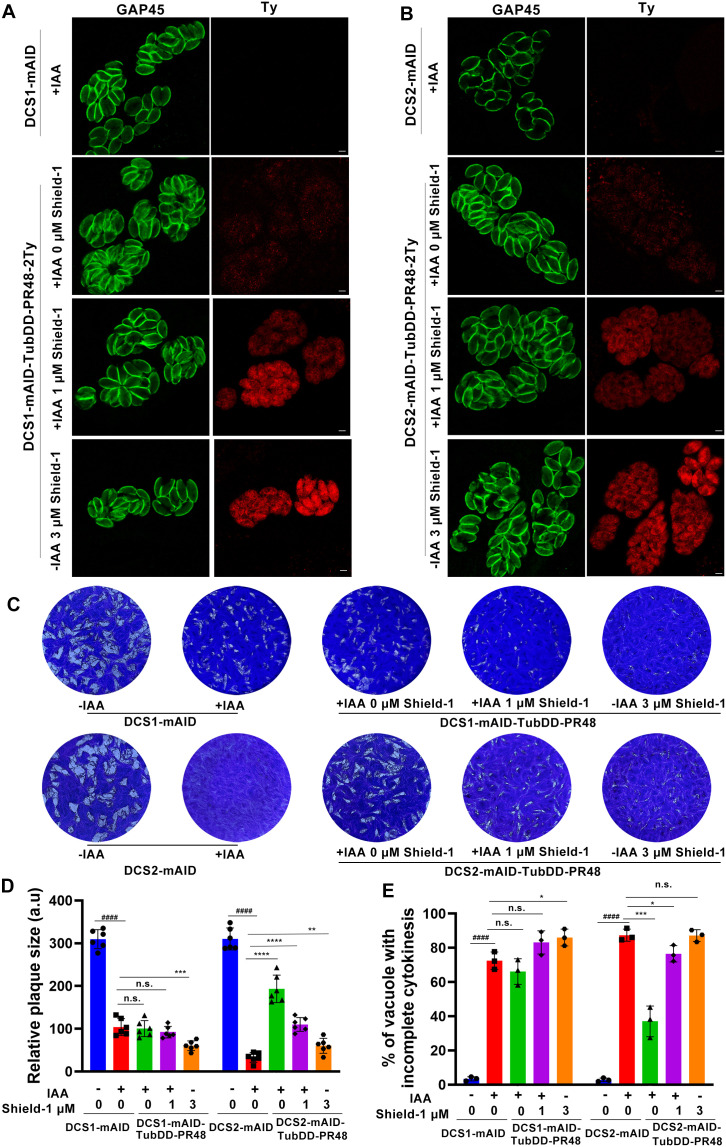
Overexpression of TgPR48 partially compensates for the depletion of DCS2 but not DCS1. (A). Immunofluorescence analysis of the DCS1-mAID-TubDD-PR48-2Ty strain showing that overexpression of TgPR48 cannot compensate for the depletion of DCS1. Infected cells were fixed 32 h post-infection and stained with anti-GAP45 (green) and anti-Ty (red) antibodies to evaluate daughter cell emergence. Scale bar: 2 µm. (B). Immunofluorescence analysis of the DCS2-mAID-TubDD-PR48-2Ty strain showing that overexpression of TgPR48 partially compensates for the depletion of DCS2. Infected cells treated with IAA or/ and Shield-1 were fixed 32 h post-infection and stained with anti-GAP45 (green) and anti-Ty (red) antibodies to evaluate daughter cell emergence. Scale bar: 2 µm. (C). Representative images of plaque assays of the indicated strains cultured under standard conditions for 7 days, with or without IAA or Shield-1 treatment. The images shown are representative of three independent experiments. (D). Quantification of plaques formed by the indicated strains cultured for 7 days under standard conditions with or without IAA. Data represent the mean ± SD from three independent experiments. Statistical significance was determined using a two-tailed, unpaired *t*-test. Compared with DCS1-mAID without IAA. ^####^*P* < 0.0001; Compared with DCS1-mAID with IAA. ***P = 0.0008; n.s., not significant. Compared with DCS2-mAID without IAA. ^####^*P* < 0.0001; Compared with DCS2-mAID with IAA. ****P < 0.0001; **P = 0.0096; n.s., not significant. (E). Quantification of vacuoles containing parasites with defective daughter cell emergence. Vacuoles with two or more mature daughter parasites enclosed within the same plasma membrane were quantified. Data represent the mean ± SD from three independent experiments. Statistical significance was determined using a two-tailed, unpaired *t*-test. Compared wi*t*h DCS1-mAID without IAA. ^####^*P* < 0.0001; Compared with DCS1-mAID with IAA. *P = 0.0293. n.s., not significant. Compared with DCS2-mAID without IAA. ^####^*P* < 0.0001; Compared with DCS2-mAID with IAA. ***P = 0.0008; *P = 0.0338; n.s., not significant.

The observation that depletion of DCS1 or DCS2 disrupts TgPR48 localization suggests that TgPR48 function may be influenced by these proteins. To assess whether TgPR48 overexpression could compensate for the loss of DCS1 or DCS2, we generated DCS1-mAID and DCS2-mAID strains, each complemented with a regulatable TgPR48 fused to a C-terminal 2Ty tag controlled by the FKBP degradation domain (DD) system. In these strains, TgPR48 undergoes constitutive degradation after translation, but its stability can be restored upon addition of Shield-1, enabling controlled accumulation of TgPR48 in the parasite [49]. Interestingly, under the DD system, overexpressed TgPR48 exhibited diffuse cytoplasmic localization and failed to accumulate at the DCS structure (Figs 7A, 7B, and S7A).

In the DCS1-mAID-TubDD-PR48–2Ty strain, TgPR48 overexpression did not rescue the defects associated with DCS1 depletion. In contrast, in the DCS2-mAID-TubDD-PR48–2Ty strain, TgPR48 overexpression induced by 1 μM Shield-1 partially restored the phenotypic defects observed upon DCS2 depletion, as demonstrated by plaque assays (Fig 7C, 7D) and fluorescence imaging (Fig 7A, 7B, 7E). These findings suggest that while functional loss of DCS2 can be partially compensated by elevated TgPR48 expression, defects resulting from DCS1 depletion are not similarly rescued. On the other hand, excessive TgPR48 expression induced by 3 μM Shield-1 in the DCS1-mAID or DCS2-mAID strains (without IAA treatment) impaired parasite growth and reduced daughter cell emergence. This indicates that elevated TgPR48 levels and the resultant hyperphosphorylation may hinder *T. gondii* proliferation (Fig 7).

Importantly, the parental RH, DCS1-mAID, and DCS2-mAID strains without the TgPR48 overexpression system did not exhibit defects in daughter cell emergence after 3 μM Shield-1 treatment, suggesting that these phenotypes are a direct consequence of TgPR48 overexpression rather than Shield-1 treatment itself (S7B Fig). Interestingly, low-level TgPR48 expression occurred in the absence of Shield-1 due to leaky activity of the DD system (S7A Fig). This residual expression was able to partially compensate for the loss of DCS2 (Fig 7). In contrast, overexpression of DCS2 in TgPR48-depleted parasites did not rescue the defects caused by TgPR48 depletion (S7C Fig). Furthermore, DCS2 overexpression in PR48-mAID parasites without IAA treatment did not disrupt daughter cell emergence, reinforcing the notion that DCS2’s functional role is distinct from that of TgPR48 (S7C Fig). These results suggest that while the PP2A-2 holoenzyme may dephosphorylate multiple substrates, including DCS1 and DCS2, altering their phosphorylation status alone does not significantly impact their function. This points to the possibility of additional unidentified phosphorylation sites or other regulatory mechanisms that govern their activity.

## Discussion

**Fig 8:**
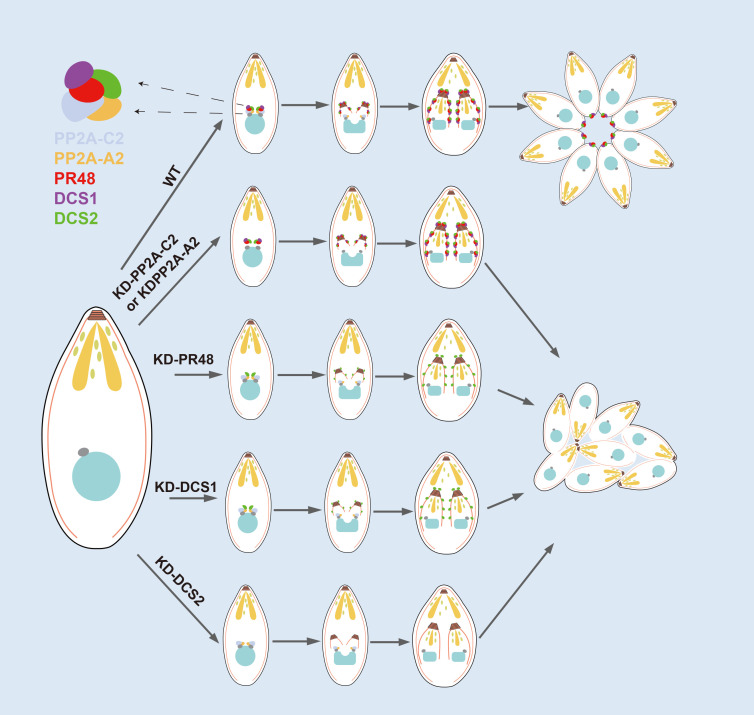
Role of the PP2A-2 holoenzyme and DCS proteins in regulating daughter cell emergence in *Toxoplasma gondii.* This schematic illustrates the involvement of the PP2A-2 holoenzyme, potentially comprising PP2A-A2, PP2A-C2, and TgPR48, as well as two potential substrate DCS proteins, in the regulation of daughter cell emergence during the division of tachyzoites. Early in division, the PP2A-2 holoenzyme and DCS proteins localize to the outer core of the centrosome after centrosome duplication. As the inner membrane complex elongates, PP2A-A2 and PP2A-C2 are downregulated and nearly undetectable in the C phase. In contrast, TgPR48 and the DCS proteins accumulate at the daughter cell buds. Upon daughter cell emergence, TgPR48 and DCS proteins localize to the basal pole of the parasite. Depletion of PP2A-C2 or PP2A-A2 does not affect DCS protein localization; however, depletion of PP2A-C2 disrupts PP2A-A2 localization, and depletion of PP2A-A2 affects PP2A-C2 localization. Conversely, depletion of TgPR48 or DCS proteins does not impact the localization of PP2A-A2 or PP2A-C2. Interestingly, there are interdependencies in the localization of TgPR48 and DCS proteins: depletion of TgPR48 disrupts DCS1 localization without affecting DCS2; depletion of DCS1 alters TgPR48 localization without affecting DCS2; and depletion of DCS2 affects both TgPR48 and DCS1 localization. Overexpression of TgPR48 partially rescues the defects in daughter cell emergence observed in DCS2-depleted parasites.

The pathogenesis of apicomplexan infections is intricately linked to the rapid replication of the parasite’s asexual stages, with cytokinesis playing a pivotal role in this process. Cytokinesis, the final and highly coordinated step of cell division, necessitates precise membrane delivery and assembly at the cleavage furrow, a task essential for successful daughter cell separation [7–10]. Within the Apicomplexa phylum, *T. gondii* tachyzoites stand out as an ideal model for dissecting the structural and molecular mechanisms governing cell division, due to their ease of culture and genetic manipulability [50]. In this study, we reveal a critical role for the PP2A-2 holoenzyme in regulating the final stages of *T. gondii* tachyzoite cell division. Disruption of PP2A-2 and its substrates, DCS1 and DCS2, impairs daughter cell emergence, arresting cytokinesis at a late stage (Fig 8).

Unlike most eukaryotic cells, which undergo binary fission with cytokinesis mediated by an actin/myosin-based contractile ring in animal cells or a phragmoplast in plants [51–53], apicomplexan parasites replicate through a distinctive process in which daughter cells assemble within the mother cell. This unique mechanism combines ancient and evolved elements specifically adapted to enable apicomplexan proliferation across diverse host environments [7–11]. Plasma membrane synthesis and trafficking are essential for cytokinesis, and previous research has demonstrated the crucial role of fatty acid synthase type II in generating the lipid substrates necessary for parasite division. Disruption of FASII results in defective cytokinesis and the formation of ‘tethered’ daughter cells, a phenotype that can be partially rescued by the addition of exogenous fatty acids [54]. Similarly, vesicle-mediated membrane delivery is crucial for successful division, with defects in Rab11A or AP1, key components of vesicle trafficking, impairing pellicle formation and preventing daughter cell separation [55–58]. Despite these advances, little is known about the molecular mechanisms that facilitate the emergence of daughter cells from the mother cell beyond vesicular transport.

The PP2A holoenzyme is a highly conserved phosphatase complex across eukaryotes, known to regulate a vast array of cellular processes, including DNA replication and cell cycle progression [33,34]. Our previous work demonstrated that TgPR48, a homolog of the human PP2A regulatory subunit PR48, dynamically localizes to the daughter cell scaffold throughout the replication cycle [35]. Depletion of TgPR48 results in defective cytokinesis, with daughter cells failing to separate or acquire plasma membranes. This disrupts rosette formation, leading to tethered parasites.

Further investigation revealed that key cellular processes such as centrosome and apicoplast replication proceeded normally, and the integrity of the inner membrane complex was preserved in TgPR48-depleted parasites. Structural elements like the basal complex and apical polar ring also assembled as expected. However, their functionality was compromised during cytokinesis. Defects in late-stage daughter cell segregation were observed at the residual body and remnants of the mother cell’s conoid, pointing to a failure in the final stages of daughter cell formation. Given the essential role of the glideosome, a myosin-actin motor complex involved in motility and host cell invasion [41], we also examined its core components (MLC1, GAP45) alongside the plasma membrane marker SAG1. TgPR48 depletion disrupted the link between the plasma membrane and inner membrane complex, highlighting its crucial role in orchestrating cytokinesis. Interestingly, while TgPR48-depleted parasites retained the ability to egress upon calcium ionophore stimulation, they exhibited defects in motility and host cell invasion. These defects appear to be secondary consequences of failed cytokinesis rather than direct effects of TgPR48 loss, reinforcing its central role in daughter cell formation.

Our findings led us to hypothesize that TgPR48 functions as part of a phosphatase complex. Among its potential interactors, PP2A-C2 stood out, exhibiting a unique localization pattern. This catalytic subunit of the PP2A-2 holoenzyme primarily localized to the centrosomal core but transiently co-localized with TgPR48 during early S phase. Sequence analysis confirmed that PP2A-C2 retains six conserved residues essential for its phosphatase activity. Depletion of PP2A-C2 recapitulated the phenotype seen with TgPR48 depletion, causing a failure in daughter cell emergence.

Leveraging the high sensitivity and throughput of the 10X proteomics platform [59], we identified 5,518 proteins across four biological replicates, surpassing previous proteomic datasets in scope. Proteomic analysis confirmed the depletion of PP2A-C2 and identified PP2A-A2 as significantly downregulated. Localization and structural analyses of PP2A-A2 revealed 15 tandem HEAT repeats, forming a horseshoe-shaped structure reminiscent of known PP2A scaffolding subunits. Reciprocal depletion of PP2A-C2 and PP2A-A2 disrupted their respective localizations, underscoring their functional interdependence. Together, our data suggest that the PP2A-2 holoenzyme in *T. gondii* is composed of three subunits: TgPR48 (the regulatory B subunit), PP2A-C2 (the catalytic C subunit), and TGME49_229350 (the scaffolding A subunit), which together form a dynamic, regulatory complex.

Our previous studies revealed that the PP2A-1 holoenzyme, involved in starch metabolism and bradyzoite differentiation, is conserved among Coccidia but absent in *Plasmodium falciparum* [35]. In contrast, PP2A-2, particularly PP2A-C2, is conserved in *P. falciparum*, suggesting a shared mechanism of cytokinetic regulation [30]. However, we found that PfPP2A-C could not rescue the defects induced by TgPP2A-C2 depletion, likely due to differences in their division modes. *T. gondii* forms its plasma membrane post-IMC maturation, whereas *P. falciparum* couples these processes [60]. This underscores the evolutionary divergence of PP2A-2 function across Apicomplexa, highlighting the need for further investigation.

Phosphoproteomic analysis following PP2A-C2 depletion revealed widespread changes in phosphorylation, with 616 sites upregulated across 495 proteins and 641 sites downregulated across 505 proteins. Some downregulated sites may reflect compensatory mechanisms, possibly through activation of alternative phosphatases or inhibition of specific kinases. To uncover the downstream effectors of PP2A-2, we integrated TurboID proximity labelling with single-cell cycle expression data, focusing on DCS1 and DCS2. Depletion of either protein impaired daughter cell emergence, supporting their roles in late-stage cytokinesis. Interestingly, functional rescue experiments using phosphomimetic and non-phosphorylatable mutants of DCS1 and DCS2 revealed that the tested phosphorylation sites are likely redundant or dispensable, suggesting a complex network of phosphorylation-based regulation at play.

Depletion of DCS1 or DCS2 also disrupted TgPR48 localization, and overexpression of TgPR48 in a DCS2-depleted background partially rescued the cytokinesis defect, implying a compensatory mechanism involving DCS1 or another substrate. Interestingly, Marq et al. independently identified DCS1 and DCS2 as critical players in cytokinetic abscission but did not explore their relationship with the PP2A-2 holoenzyme. Their knockdown strategy, which targeted PP2A-C2 and PP2A-A2 at the transcriptional level, may account for the milder phenotypes observed compared to the more severe effects following TgPR48 depletion [48].

In conclusion, our study highlights the essential role of the PP2A-2 holoenzyme, composed of TgPR48, PP2A-C2, and PP2A-A2, in regulating daughter cell emergence during *T. gondii* division. Disruption of any subunit results in severe cytokinetic defects, preventing proper segregation of daughter cells. Phosphoproteomic analysis identifies DCS1 and DCS2 as potential PP2A-2 substrates, although their exact roles in phosphorylation-dependent regulation remain to be fully elucidated. These findings offer new insights into the molecular machinery driving apicomplexan cytokinesis and suggest promising avenues for future research into the evolutionary conservation and divergence of this regulatory mechanism across related parasites, particularly *Plasmodium* spp.

## Materials and methods

### Parasites and host cell maintenance

The parasites used in this study, including RHΔ*ku80*Δ*hxgprt*::TIR1–3Flag (RH) and PruΔ*ku80*Δ*hxgprt*::TIR1–3Flag (Pru), were cultured in confluent monolayers of human foreskin fibroblasts (HFFs) grown in Dulbecco′s Modified Eagle Medium (DMEM) supplemented with 2% fetal bovine serum (FBS), 100 mg/ml streptomycin, and 10 units/ml penicillin at 37°C with 5% CO_2_. Prior to infection, HFFs were cultured to confluence in DMEM with 10% FBS at 37°C in 5% CO_2_ [35,61]. Fresh tachyzoites were released from heavily infected host cells by passing them through a 27-gauge needle, followed by filtration through a 3-µm polycarbonate membrane.

### Primers and plasmids

Plasmid construction was performed either by site-directed mutagenesis of existing plasmids using the Q5 Site-Directed Mutagenesis Kit (New England Biolabs) or by DNA fragment assembly with the ClonExpress MultiS One Step Cloning Kit (Vazyme, China). Detailed information on the primers and plasmids used in this study is available in S6 Table.

### Construction of transgenic parasite strains

CRISPR-Cas9 tagging technology was used to generate transgenic parasite strains with endogenous protein fusions at either the C- or N-terminus [35,61]. For C-terminal tagging, CRISPR-Cas9 plasmids targeting the 3′-UTR of the gene of interest, downstream of the stop codon, were co-transfected with amplicons containing short homology regions. These amplicons included mAID-2Ty, mAID-6HA, TurboID-4Ty, or 2Ty tag, along with a drug selection cassette, into the respective parasite strain [40]. For N-terminal tagging, CRISPR-Cas9 plasmids targeting the 5′-UTR, upstream of the start codon, were co-transfected with amplicons derived from generic tagging plasmids, such as pN-DHFR-pPP2A-C2-6HA-mAID, pN-DHFR-pPP2A-C2-2Ty or pN-DHFR-pMORN1–2Ty [62]. The complementation line was generated by inserting a fragment containing the endogenous promoter, the coding sequence tagged with 2Ty at either the C- or N-terminus, and the *hxgprt* 3′-UTR into the *uprt* locus. Transfectants were selected using 3 μM pyrimethamine, 25 μg/ml mycophenolic acid, 25 μg/ml 6-xanthine, or 20 µM chloramphenicol, and single clones were isolated via limiting dilution. Tagging success was confirmed through PCR, DNA sequencing, and IFA. For conditional knockdown, parasites were incubated with 500 µM auxin (IAA) (1:1000 dilution), while control treatments were performed with 0.1% ethanol [40].

### Immunofluorescence assay and Western blotting

IFA was performed on HFF monolayers pre-seeded on coverslips, allowing parasites to infect the cells. Infected samples were fixed in 4% paraformaldehyde (PFA) for 20 min, washed three times with phosphate-buffered saline (PBS), and permeabilized with 0.1% Triton X-100 for 15 min. Sample were then blocked in PBS containing 5% bovine serum albumin (BSA) for 1 h at 37°C. Primary antibodies were applied and incubated overnight at 4°C or for 2 h at 37°C, followed by five washes with PBS and a 2 h incubation with secondary antibodies. Nuclei were counterstained with 4’,6-diamidino-2-phenylindole (DAPI). After five additional PBS washes, imaging was conducted using a Leica TCS SP8 confocal microscope (Leica Microsystems, Germany).

For Western blotting, freshly lysed parasites were syringe-filtered, collected by centrifugation, washed twice in PBS, and pelleted at 2000 × *g* for 10 min at 4°C. Cell pellets were resuspended in RIPA buffer supplemented with protease and phosphatase inhibitors, incubated on ice for 45 min, and then centrifuged at 12,000 ×* *g** for 10 min at 4°C to collect the supernatant. Supernatants were then mixed with 4 × Laemmli buffer, heated at 100°C for 10 min, and separated on 8% SDS-PAGE gels. Proteins were transferred to nitrocellulose membranes using a wet transfer system (Bio-Rad). Membranes were blocked with 5% non-fat milk in TBS/0.2% Tween 20, probed with primary antibodies followed by secondary antibodies, and washed thoroughly. Protein detection was carried out using Pierce ECL substrate, and chemiluminescent signals were captured with the ChemiDoc XRS+ imaging system (Bio-Rad, USA). All primary antibodies used in this study were either generated in our laboratory and validated in previous publications or sources commercially. Detailed information is provided in S6 Table.

### Plaque assay

Plaque assays were performed in 12-well plates by inoculating confluent HFF monolayers with equal numbers of freshly released parasites. The parasites were allowed to grow for 7 days in the presence or absence of 500 µM IAA. Following incubation, samples were fixed with 4% PFA in PBS for 20 min and stained with 0.2% crystal violet for 20 min at room temperature. After washing with PBS, plaque areas were manually delineated and quantified using ImageJ software. All plaque assays were performed in at least three independent biological replicates.

### Invasion assay

A two-color immunofluorescence assay was used to assess tachyzoite host cell invasion [63,64]. For the invasion assay, tachyzoites were cultured for 48 h with or without IAA, harvested by scraping, passed through a 27-gauge needle, and allowed to infect HFFs for 30 min. Freshly isolated tachyzoites were suspended in DMEM, with or without IAA, and added to HFF monolayers on coverslips in 24-well plates. The cultures were incubated for 30 min at 37°C. After fixation with 4% PFA, extracellular parasites were stained with mouse anti-SAG1 and the corresponding secondary antibody. Samples were permeabilized with 0.1% Triton X-100, and all parasites were stained with rabbit anti-GAP45 and the secondary antibody. Parasites were classified as extracellular (GAP45^+^SAG1^+^) or intracellular (GAP45^+^SAG1^−^) using confocal microscopy. Each invasion assay was performed in triplicate, with at least 15 fields counted per replicate.

### Replication assay

Freshly isolated tachyzoites were inoculated onto HFF monolayers in 12-well tissue culture plates and incubated for 1 h. After washing, the infected cultures were incubated in fresh medium, with or without IAA. At 24 h post-infection, the samples were fixed and stained with rabbit anti-IMC1 antibody. The number of parasites per PV was quantified using fluorescence microscopy. At least 150 vacuoles were analyzed across three biological replicates to determine the number of tachyzoites per vacuole.

### Ionophore-induced egress assay

Freshly isolated tachyzoites were used to infect HFF monolayers in 12-well plates for 36–40 h, in the presence or absence of IAA, until most PVs contained a similar number of tachyzoites. Cells were then treated with 3 µM A23187 or DMSO (control) in DMEM at 37°C for 3 min, followed by fixation and permeabilization. PVs were labeled with anti-GRA5, and parasites were stained with anti-SAG1, followed by secondary antibody staining to distinguish between intact and lysed PVs [61,64]. Egress was quantified by counting lysed PVs, with at least 100 PVs examined per plate. The assay was performed independently three times.

### Transmission electron microscopy

Freshly isolated tachyzoites were allowed to infect HFF monolayers for 24 h. Infected samples were then fixed with 2.5% glutaraldehyde and 0.5% tannic acid in 0.1 M sodium cacodylate buffer, followed by post-fixation in 1% osmium tetroxide and 1.5% potassium ferricyanide. The samples were subsequently dehydrated using a graded ethanol series and embedded in LX112 resin. Ultrathin sections were prepared with an Ultracut UCT, stained with uranyl acetate and lead citrate, and examined using a Hitachi HT7700 electron microscope at 80 kV [35].

### Bradyzoites differentiation

Confluent HFF cells were infected with freshly egressed tachyzoites for 2 h under normal conditions (pH 7.4 DMEM with 2% FBS) in the presence or absence of IAA [35,61]. The medium was then replaced with alkaline RPMI-HEPES (pH 8.2) containing 2% FBS, with or without IAA. The cultures were maintained at 37°C in ambient CO_2_ (~ 0.03%), with daily medium changes to sustain the alkaline pH. After 3 days, parasites were stained with anti-IMC1 to visualize all the parasites, and the parasite cyst wall was labeled using FITC-conjugated *Dolichos biflorus* lectin (DBL). Bradyzoites differentiation was assessed by calculating the percentage of DBL-positive PVs, with at least 100 PVs analyzed from three biological replicates.

### Microneme secretion assay

To assess microneme secretion, approximately 2 × 10^7^ PR48-mAID tachyzoites, treated with or without IAA for 48 h, were harvested by centrifugation at 1,500 ×* *g** to remove residual DMEM. Parasites were resuspended in 200 µL of fresh DMEM. For stimulation, 200 µL of DMEM supplemented with 2% ethanol and 6% FBS was added to induce MIC2 secretion. In parallel, control samples received an equal volume of DMEM without stimulants. All samples were incubated at 37 °C with 5% CO₂ atmosphere for 3 min, then immediately placed on ice for 5 min to halt secretion. Samples were centrifuged at 2,000 × *g* to separate supernatants and pellets. The pellet fraction served as the loading control, while the supernatant was clarified to isolate 250 µL of the excreted-secreted antigen (ESA) fraction. Pellets were lysed in 100 µL of RIPA buffer supplemented with EDTA and protease inhibitors for 1 h on ice. Both ESA and pellet lysates were denatured by boiling in 4 × loading buffer and analyzed via SDS-PAGE followed by immunoblotting. The experiment was conducted in three independent biological replicates. Primary antibodies used included rabbit anti-MIC2 (1:1000), mouse anti-GRA1 (1:1000), and rabbit anti-aldolase (1:500).

### Affinity capture of biotinylated proteins

HFF monolayers were infected with either parental tachyzoites or TurboID-tagged parasites for 36 h, followed by growth in medium containing 150 μM biotin for an additional 4 h. Intracellular parasites from large vacuoles were harvested by manual scraping, washed with PBS, and lysed in RIPA buffer supplemented with protease and phosphatase inhibitors. The lysate was incubated with streptavidin beads (Pierce) overnight at 4°C with gentle agitation. After extensive washing, the beads were collected, and 5% of each sample was boiled in Laemmli sample buffer. Eluted proteins were analyzed by Western blotting using streptavidin-HRP, while the remaining sample was prepared for mass spectrometry analysis [65]. This experiment was performed in triplicate.

### Mass spectrometry analysis of immunoprecipitated and biotinylated proteins

Eluted proteins were resolved by SDS-PAGE and stained with Coomassie blue R250. Protein bands were carefully excised, frozen, and subjected to vacuum drying. The dried gel slices were rehydrated, finely minced, and processed to remove the stain and SDS via reduction, alkylation, and washing steps. The samples were subsequently digested with trypsin, dried again, and reconstituted in a solution of 2.5% acetonitrile and 0.1% formic acid. After centrifugation at 20,000 *g* for 10 min, the supernatant was injected into a Thermo UltiMate 3000 UHPLC system. Peptides were separated on a self-packed C18 column (75 μm ID, 3 μm particle size, 25 cm length) at a flow rate of 300 nL/min using a gradient of 98% acetonitrile and 0.1% formic acid: 5% for 0–5 min, 5%-25% for 5–45 min, 25%-35% for 45–50 min, 35%-80% for 50–52 min, 80% for 52–54 min, and 5% for 54–60 min. Separated peptides were ionized by nanoESI and analyzed on a Q-Exactive HF X mass spectrometer in DDA mode. MS1 scans were acquired at a resolution of 60,000 over a 350–1,500 m/z range, with the top 30 ions (charge states 2+ to 6+) selected for HCD fragmentation in MS2 scans at a resolution of 15,000. Dynamic exclusion was set to 30 sec, with AGC targets of 3E6 for MS1 and 1E5 for MS2. Raw protein mass spectrometry data were analyzed using MaxQuant (v1.6.1.0) against the *T. gondii* GT1 database from ToxoDB (https://toxodb.org) [66]. The iBAQ values for each protein were calculated using BGI′s proprietary software to extract peptide XIC and determine the peak area [67]. Peptide and protein identifications were filtered with a 1% false discovery rate (FDR). Reversed sequences, contaminants, and proteins identified by only a single modification site were excluded from the final dataset. Preliminary screening was conducted by selecting peptides from the treated group that exhibited a ≥ 2-fold upregulation compared to the control group.

### Phosphoproteomics studies

#### Protein extraction.

Intracellular parasites from large vacuoles, with or without IAA treatment, were collected by manual scraping, washed with PBS, and lysed in buffer containing protease and phosphatase inhibitors. The lysates were sonicated on ice for 3 min using a high-intensity ultrasonic processor. After centrifugation at 12,000 × *g* at 4°C for 10 min to remove debris, the supernatant was collected, and protein concentration was measured using a BCA kit.

#### Trypsin digestion.

The sample was treated with 20% (m/v) trichloroacetic acid to precipitate proteins, vortexed, and incubated for 3 h at 4°C. The precipitate was collected by centrifugation at 4500 × *g* for 10 min at 4°C, washed three times with pre-cooled acetone, and air-dried. The protein pellet was redissolved in 200 mM TEAB and sonicated. Proteins were digested with trypsin at a 1:50 trypsin-to-protein ratio overnight. The sample was reduced with 5 mM dithiothreitol at 56°C for 30 min, followed by alkylation with 11 mM iodoacetamide for 15 min in the dark at room temperature. Peptides were desalted using a Strata X SPE column.

#### Affinity enrichment.

Peptide mixtures were incubated with IMAC microspheres in a loading buffer consisting of 50% acetonitrile and 0.5% acetic acid under continuous agitation. To remove non-specifically bound peptides, the microspheres were washed sequentially with 50% acetonitrile/0.5% acetic acid and 30% acetonitrile/0.1% trifluoroacetic acid. Phosphopeptides were eluted by adding 10% NH₄OH elution buffer with agitation. The supernatant containing the enriched phosphopeptides was collected, lyophilized, and subjected to LC-MS/MS analysis.

#### Mass spectrometry analysis.

Tryptic peptides were dissolved in solvent A (0.1% formic acid in water) and directly loaded onto a homemade reversed-phase analytical column (15 cm in length, 100 μm i.d.). Peptide separation was achieved using a gradient of solvent B (0.1% formic acid, 80% acetonitrile), ranging from 2.0% to 99.0% over 22.6 min, at a constant flow rate of 400 nL/min on a Vanquish Neo UPLC system (Thermo Fisher Scientific). The separated peptides were then analyzed on an Orbitrap Astral mass spectrometer equipped with a nano-electrospray ion source, operating at an electrospray voltage of 1900 V. Precursor ions were scanned at a resolution of 240,000 over a range of 380–980 m/z, while fragment ions were analyzed at a resolution of 80,000 using HCD fragmentation with a 25% normalized collision energy. The AGC target was set to 500%, with a maximum injection time of 3 ms. MS/MS data were processed using DIA-NN software (v.1.8) [68] and searched against the *T. gondii* GT1 database from ToxoDB (https://toxodb.org) [66], along with a reverse decoy database. A false discovery rate (FDR) of < 1% was applied.

### Statistical analyses

Statistical analyses were conducted using Prism software version 10.0 (GraphPad Software Inc., CA, USA). Detailed information on the number of biological replicates, observations, error bar definitions, and statistical tests used can be found in the figure legends. A *P* value of < 0.05 was considered statistically significant. All microscopy images are representative of at least two independent experiments, with consistent results obtained across all experiments.

## Supporting information

S1 Fig Functional characterization of the TgPR48 subunit in *Toxoplasma gondii.* **(A).** Schematic representation of endogenous tagging at the C-terminus of the gene of interest (GOI). PCR1 (~ 500–600 bp) was used to detect the indicated strains with a short extension time (15 s) to confirm modification of the C-terminus. Successful tag insertion was validated with PCR2 (~ 600–700 bp) and DNA sequencing. **(B).** Immunofluorescence analysis of intracellular RH::PR48-mAID-6HA and ComPR48-mAID-6HA parasites treated with or without IAA for 24 h. Parasites were stained with anti-IMC1 (green) and anti-HA (red) or anti-Ty (red) antibodies. Scale bar: 2 µm. **(C).** Western blot analysis of total protein extracts from the RH::PR48-mAID-6HA strains treated with IAA for different durations. Degradation of the tagged protein via the mAID system was confirmed using an anti-HA antibody, and aldolase (ALD) was used as the loading control. **(D).** Microneme secretion assays revealed reduced MIC2 secretion in TgPR48-depleted parasites after induction with 1% ethanol and 3% FBS. The stimulated group (+) was incubated in DMEM containing 1% ethanol and 3% FBS, whereas the control group (−) was treated with DMEM alone. MIC2 levels in the supernatant (secreted fraction) and cell lysate (cellular fraction) were analyzed by Western blotting using rabbit anti-MIC2 antibody. GRA1 and ALD were used as controls for the secreted and cellular fractions, respectively, detected with mouse anti-GRA1 and rabbit anti-ALD antibodies. **(E).** Immunofluorescence analysis was performed on RH::PR48-mAID-6HA parasites following treatment with or without IAA for 32 h. Parasites were stained with anti-SAG1 (green) or anti-MLC1 (green) and anti-IMC1 (red) antibodies. Scale bar: 2 µm. **(F).** Immunofluorescence analysis of RH::PR48-mAID-6HA parasites treated with or without IAA for 12 h. Parasites were stained with anti-IMC1 (green) and anti-GAP45 (red) antibodies. Scale bar: 2 µm. **(G).** Western blot analysis of total protein extracts from the Pru::PR48-mAID-6HA strains treated with IAA for different durations. Degradation of the tagged protein via the mAID system was confirmed using an anti-HA antibody, and ALD served as the loading control. **(H).** Depletion of TgPR48 did not affect bradyzoite differentiation. Pru::PR48-mAID-infected HFF cells were incubated in an alkaline culture medium with IAA or vehicle (control) for 3 days under CO₂-free conditions to induce bradyzoites. Parasites were stained with anti-IMC1 (red) and FITC-*Dolichos biflorus* lectin (DBL) (green) to visualize bradyzoite cyst walls. Scale bar: 2 µm. **(I).** Quantification of DBL-positive vacuoles, counting at least 100 vacuoles per condition. Data are shown as mean ± SD from three independent experiments. Statistical significance was assessed using a two-tailed, unpaired *t*-test. n.s., not significant.
(TIF)

S2 Fig Impact of TgPR48 depletion on various parasite organelles. The RH::PR48-mAID-6HA strain infecting HFF cells was cultured under normal conditions and treated with or without IAA for 24–28 h. Parasites were stained with specific antibodies targeting various organelles: anti-MIC2 (micronemes, red), anti-CPN60 (apicoplast, red), anti-GRA5 (dense granules, red), anti-ARO (rhoptries, red), anti-HSP60 (mitochondria, red), anti-SAS6–2Ty or anti-Sfi1–2Ty (outer centrosome core, red), anti-CEP250-L1-2Ty (inner centrosome core, red), anti-kinetochore Nuf2–2Ty (kinetochore, red), anti-Chromo1–2Ty (centromere, red), and anti-RNG1–2Ty or anti-RNG2–2Ty (apical polar ring, red). Yellow arrows indicate the presence of remnant mother apical polar rings. Scale bar: 2 µm.
(TIF)

S3 Fig Functional characterization of the PP2A-C2 subunit in *Toxoplasma gondii.* **(A).** Immunofluorescence analysis of the RH::PR48-TurboID-4Ty strain showing proteins in proximity to TgPR48 labeled and visualized with streptavidin conjugated with Alexa Fluor 594 after D-biotin treatment. Green: mouse anti-Ty; red: streptavidin Alexa Fluor 594 conjugate; magenta: rabbit anti-IMC1. Scale bar: 2 µm. **(B).** Western blot analysis confirming increased levels of streptavidin-HRP-labeled proteins in the RH::PR48-TurboID-4Ty strain after D-biotin induction. White arrow denotes biotinylated apicoplasts, and yellow arrow indicates proteins biotinylated by PR48-TurboID. Strep-HRP: Streptavidin-Horseradish Peroxidase conjugate; ALD: rabbit anti-aldolase. **(C).** Schematic representation of endogenous tagging at the N-terminus of the PP2A-C2 gene. Tag insertion was validated by PCR and DNA sequencing. **(D).** Immunofluorescence analysis of intracellular RH::mAID-6HA-PP2A-C2 parasites treated with or without IAA for 24 h. Parasites were stained with anti-IMC1 (red) and anti-HA (green) antibodies. Scale bar: 2 µm. **(E).** Western blot analysis of total protein extracts from RH::mAID-6HA-PP2A-C2 strains treated with IAA for varying durations. Protein degradation was confirmed with an anti-HA antibody, and ALD served as the loading control. **(F).** Immunofluorescence analysis showing the subcellular localization of 2Ty-PP2A-C2 in RH::CRK4-mAID parasites following treatment with or without IAA for 24 h, indicating that PP2A-C2 remained localized at the centrosome regardless of CRK4 depletion. Parasites were stained with anti-IMC1 (green) and anti-Ty (red) antibodies. Scale bar: 2 µm. **(G).** Functional analysis of the PP2A-C2 catalytic motif consensus sequences. Immunofluorescence analysis of PP2A-C2-depleted parasites re-expressing the wild-type or mutant versions of 2Ty-tagged PP2A-C2, or 2Ty-tagged PfPP2A-C under the control of native TgPP2A-C2 promoters. Parasites were cultured under normal conditions for 28 h, followed by immunofluorescence detection with anti-IMC1 (green) and anti-Ty (red) antibodies. Scale bar: 2 µm. **(H).** Western blot analysis showing reduced protein levels of mutant 2Ty-PP2A-C2 constructs in IAA-treated parasites, whereas expression of 2Ty-PfPP2A-C confirmed successful protein production. The presence of mutant 2Ty-PP2A-C2 or 2Ty-PfPP2A-C was detected using an anti-Ty antibody, with ALD serving as the loading control.
(TIF)

S4 Fig Functional characterization of the PP2A-A2 subunit in *Toxoplasma gondii.* **(A).** Schematic representation of the PP2A-A2 gene model highlighting the HEAT repeat-containing domain**. (B).** Immunofluorescence analysis of intracellular RH::PP2A-A2-mAID-6HA and ComPP2A-A2-mAID-6HA parasites treated with IAA for 24 h. Parasites were stained with anti-IMC1 (green) and anti-HA (red) or anti-Ty (red) antibodies. Scale bar: 2 µm. **(C).** Western blot analysis of total protein extracts from the RH::PP2A-A2-mAID-6HA strains treated with IAA for varying durations. Degradation of the tagged protein was confirmed via the mAID system using an anti-HA antibody, with aldolase (ALD) as the loading control. **(D).** Structural model of the PP2A-2 holoenzyme generated using AlphaFold 3. **(E).** Western blot analysis of total protein extracts from the indicated strains treated with or without IAA for 24 h showed a reduction in one subunit when the other was depleted, indicating a functional link between PP2A-C2 and PP2A-A2. 2Ty-PP2A-C2 and PP2A-A2-2Ty proteins were detected using an anti-Ty antibody, with ALD serving as the loading control. **(F).** Quantification of fold changes in relative protein levels of PP2A-C2 in PP2A-A2-mAID parasites, and PP2A-A2 in mAID-PP2A-C2 parasites, was performed by comparing samples cultured in the absence or presence of IAA across three independent replicates. Western blotting band intensities were quantified using ImageJ software, and protein levels were normalized to the ALD signal, which served as a loading control. **(G).** Immunofluorescence analysis of the localization of TgPR48, PP2A-A2, and PP2A-C2 after depletion of mAID-tagged proteins. Strains were treated with IAA for 24 h to induce protein depletion. Ty-tagged proteins were detected using an anti-Ty antibody, and IMC1 was used for parasite visualization. Scale bar: 2 µm. **(H).** Immunofluorescence analysis of the co-localization of TgPR48 and PP2A-C2 during the tachyzoite cell cycle. RH::mAID-6HA-PP2A-C2::PR48–2Ty infected cells were fixed 24 h post-infection and stained with anti-HA antibody to detect HA-tagged PP2A-C2 (green), anti-Ty antibody to label the 2Ty-tagged TgPR48 (red), and Hoechst dye to stain DNA (blue). Scale bar: 2 µm.
(TIF)

S5 Fig Two cell cycle-expressed genes are not involved in daughter cell emergence. **(A).** Schematic representation illustrating the identification of the potential substrates of the PP2A-2 holoenzyme. **(B).** Expression patterns of the PP2A-2 holoenzyme and four hyperphosphorylated proteins based on data from the Toxo Single Cell Atlas. **(C).** Western blot analysis of total protein extracts from the RH::DCS1-mAID-6HA strains treated with IAA for different durations. Degradation of the tagged protein via the mAID system was confirmed using an anti-HA antibody. Aldolase (ALD) served as a loading control. **(D).** Western blot analysis of total protein extracts from the RH::DCS2-mAID-6HA strain treated with IAA for different durations. Degradation of the tagged protein via the mAID system was confirmed using an anti-HA antibody. ALD served as the loading control. **(E).** Immunofluorescence analysis of mAID-6HA-tagged TGME49_260580 during the tachyzoite cell cycle. RH::TGME49_260580-mAID-6HA-infected cells were fixed 24 h post-infection and stained with anti-IMC1 antibody (green) to visualize the cell cycle, anti-HA antibody (red) to detect TGME49_260580, and Hoechst dye (blue) to stain DNA. Scale bar: 2 µm. **(F).** Immunofluorescence analysis of mAID-6HA-tagged TGME49_236560 during the tachyzoite cell cycle. RH::TGME49_236560-mAID-6HA-infected cells were fixed 24 h post-infection and stained with anti-IMC1 antibody (green) to visualize the cell cycle, anti-HA antibody (red) to detect TGME49_236560, and Hoechst dye (blue) to stain DNA. Scale bar: 2 µm. **(G)** Immunofluorescence analysis of intracellular TGME49_260580-mAID-6HA parasites treated with IAA for 24–28 h. Parasites were stained with anti-IMC1 (green) and anti-HA (red) antibodies to detect TGME49_260580, and anti-GAP45 (green) and anti-IMC1 (red) antibodies to assess defects in daughter cell emergence. Scale bar: 2 µm. **(H)** Immunofluorescence analysis of intracellular TGME49_236560-mAID-6HA parasites treated with IAA for 24–28 h. Parasites were stained with anti-IMC1 (green) and anti-HA (red) antibodies to detect TGME49_236560, and anti-GAP45 (green) and anti-IMC1 (red) antibodies to assess defects in daughter cell emergence. Scale bar: 2 µm.
(TIF)

S6 Fig Immunofluorescence analysis of the localization of PP2A-2 holoenzyme, DCS1, and DCS2 following depletion of mAID-tagged proteins. The indicated strains were treated with IAA for 24–28 h to induce depletion. Ty-tagged proteins were detected using an anti-Ty antibody, and rabbit anti-IMC1 was used to visualize the parasites. Scale bar: 2 µm.
(TIF)

S7 Fig Overexpression of DCS2 does not compensate for the depletion of TgPR48. **(A).** Western blot analysis of total protein extracts from the indicated strains treated with IAA and/or Shield-1 for 48 h. The degradation domain system effectively regulates protein expression, as demonstrated by the levels of 2Ty-tagged TgPR48 detected using an anti-Ty antibody. Aldolase (ALD) was used as the loading control. **(B).** Immunofluorescence analysis of the indicated parasites to evaluate the effect of the Shield-1. Infected cells treated with IAA and/or Shield-1 were fixed 32 h post-infection and stained with anti-GAP45 (green) and anti-IMC1 (red) antibodies to assess daughter cell emergence. Scale bar: 2 µm. **(C).** Immunofluorescence analysis of the PR48-mAID-TubDD-DCS2–2Ty strain showing that overexpression of DCS2 cannot compensate for the depletion of TgPR48. Infected cells treated with IAA and/or Shield-1 were fixed 32 h post-infection and stained with anti-GAP45 (green) and anti-Ty (red) antibodies to evaluate daughter cell emergence. Scale bar: 2 µm.
(TIF)

S1 Table Summary of mass spectrometry analysis of *Toxoplasma gondii* proteins identified from streptavidin bead–purified samples of the parental strain and the PR48-TurboID strain cultured in the presence of D-biotin. (XLSX)

S2 Table Summary of the *Toxoplasma gondii* peptides identified in mAID-6HA-PP2A-C2 parasites treated with auxin or ethanol for 48 hours, as analyzed by proteomics analysis. (XLSX)

S3 Table Summary of the significantly differentially expressed *Toxoplasma gondii* proteins identified in mAID-6HA-PP2A-C2 parasites treated with auxin or ethanol for 48 hours, as determined by proteomics analysis. (XLSX)

S4 Table Summary of the *Toxoplasma gondii* phosphopeptides identified in mAID-6HA-PP2A-C2 parasites treated with auxin or ethanol for 48 hours, as analyzed by phosphoproteomics analysis. (XLSX)

S5 Table Summary of the significantly differentially expressed *Toxoplasma gondii* phosphopeptides identified in mAID-6HA-PP2A-C2 parasites treated with auxin or ethanol for 48 hours, based on phosphoproteomics analysis. (XLSX)

S6 Table Summary of primers, plasmids and antibodies used in the study. (XLSX)

S7 Table Data that underlies this paper. (XLSX)

## Abbreviations

DAPI: 4’,6-diamidino-2-phenylindole
DBL: Dolichos bifloru*s* lectin
DCS: daughter cell scaffold
DCS1: daughter cell scaffold protein 1
DCS2: daughter cell scaffold protein 2
DMEM: Dulbecco′s Modified Eagle Medium
CDKs: cyclin-dependent kinases
FBS: fetal bovine serum
FDR: false discovery rate
GOI: gene of interest
HEAT: Huntingtin-elongation-A subunit-TOR
HFFs: human foreskin fibroblasts
IFA: Immunofluorescence analysis
IMC: inner membrane complex
mAID: mini auxin-inducible degron
PBS: phosphate buffered saline
PP2A: protein phosphatase 2A
PP6: protein phosphatase 6
PFA: paraformaldehyde
PM: plasma membrane
PV: parasitophorous vacuole
TEM: transmission electron microscopy
