# Supplementary figures and images for: The PP2A-2 holoenzyme orchestrates daughter cell emergence during cytokinesis in Toxoplasma gondii
Source: PLoS Pathog. 2025 Sep 3;21(9):e1013475. doi: 10.1371/journal.ppat.1013475 (PMC12425318; doi:10.1371/journal.ppat.1013475)

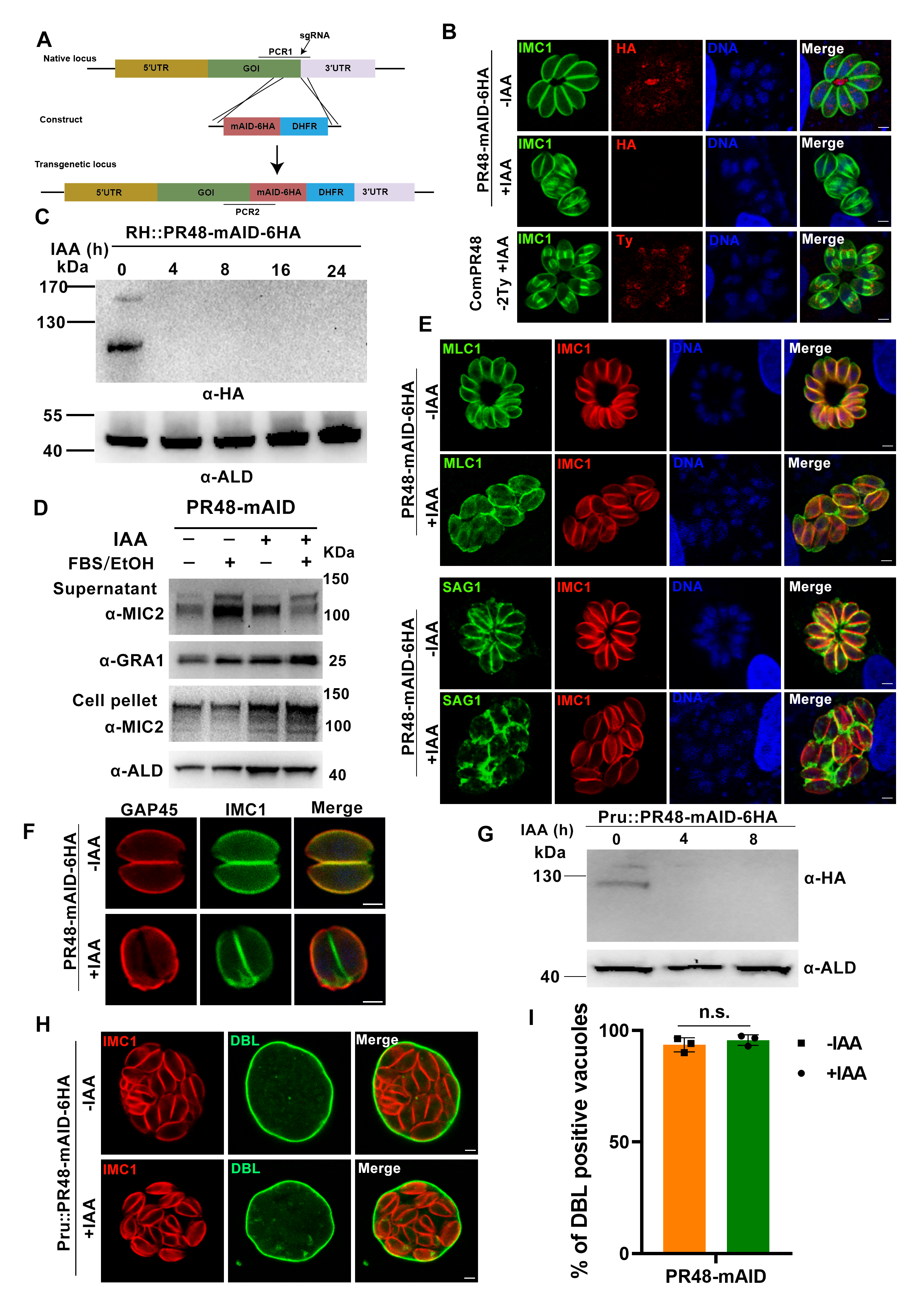

Supplement: S1 Fig — (A). Schematic representation of endogenous tagging at the C-terminus of the gene of interest (GOI). PCR1 (~ 500–600 bp) was used to detect the indicated strains with a short extension time (15 s) to confirm modification of the C-terminus. Successful tag insertion was validated with PCR2 (~ 600–700 bp) and DNA sequencing. (B). Immunofluorescence analysis of intracellular RH::PR48-mAID-6HA and ComPR48-mAID-6HA parasites treated with or without IAA for 24 h. Parasites were stained with anti-IMC1 (green) and anti-HA (red) or anti-Ty (red) antibodies. Scale bar: 2 µm. (C). Western blot analysis of total protein extracts from the RH::PR48-mAID-6HA strains treated with IAA for different durations. Degradation of the tagged protein via the mAID system was confirmed using an anti-HA antibody, and aldolase (ALD) was used as the loading control. (D). Microneme secretion assays revealed reduced MIC2 secretion in TgPR48-depleted parasites after induction with 1% ethanol and 3% FBS. The stimulated group (+) was incubated in DMEM containing 1% ethanol and 3% FBS, whereas the control group (−) was treated with DMEM alone. MIC2 levels in the supernatant (secreted fraction) and cell lysate (cellular fraction) were analyzed by Western blotting using rabbit anti-MIC2 antibody. GRA1 and ALD were used as controls for the secreted and cellular fractions, respectively, detected with mouse anti-GRA1 and rabbit anti-ALD antibodies. (E). Immunofluorescence analysis was performed on RH::PR48-mAID-6HA parasites following treatment with or without IAA for 32 h. Parasites were stained with anti-SAG1 (green) or anti-MLC1 (green) and anti-IMC1 (red) antibodies. Scale bar: 2 µm. (F). Immunofluorescence analysis of RH::PR48-mAID-6HA parasites treated with or without IAA for 12 h. Parasites were stained with anti-IMC1 (green) and anti-GAP45 (red) antibodies. Scale bar: 2 µm. (G). Western blot analysis of total protein extracts from the Pru::PR48-mAID-6HA strains treated with IAA for different duratio [file ppat.1013475.s001.tif]

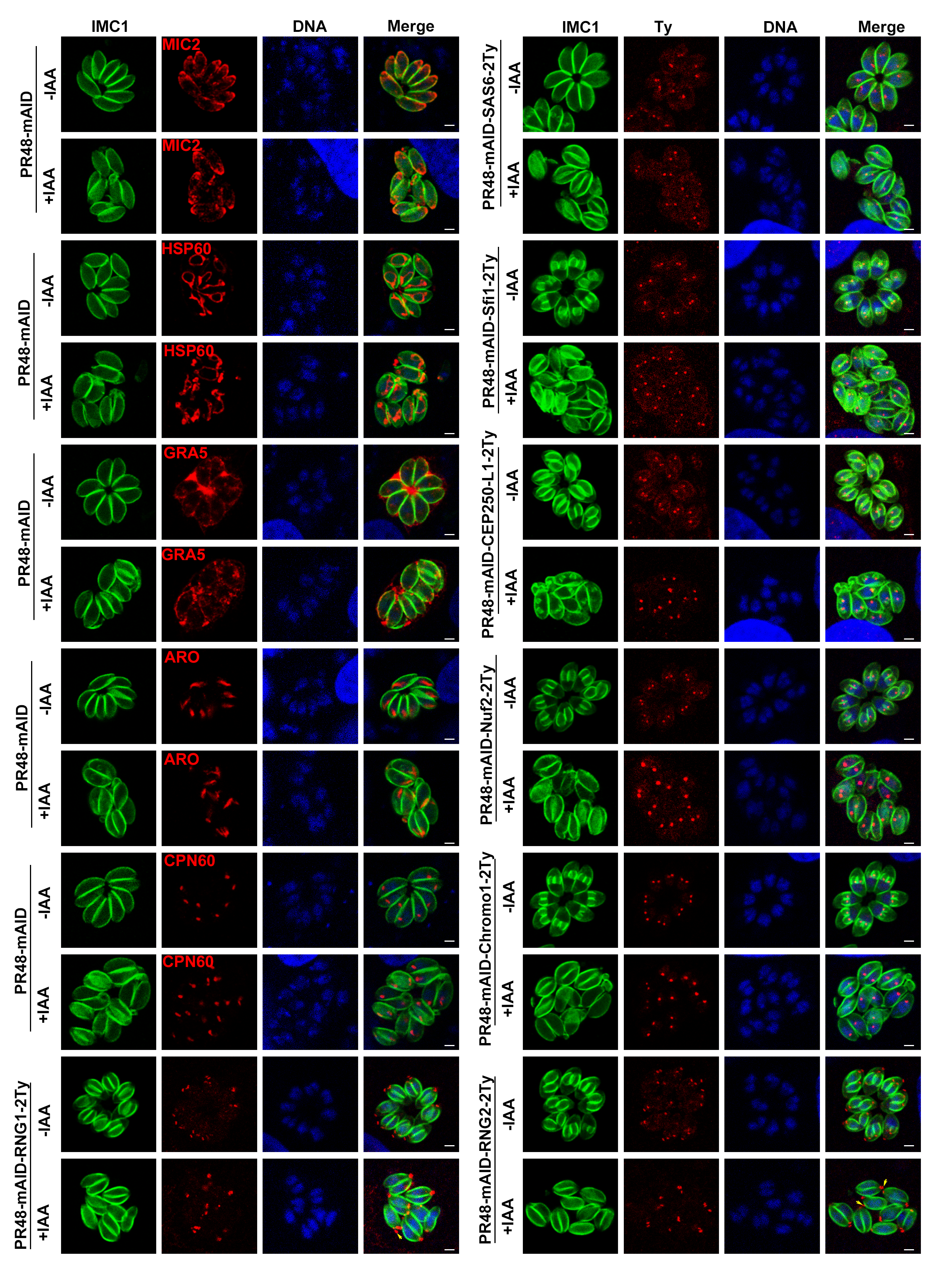

Supplement: S2 Fig — The RH::PR48-mAID-6HA strain infecting HFF cells was cultured under normal conditions and treated with or without IAA for 24–28 h. Parasites were stained with specific antibodies targeting various organelles: anti-MIC2 (micronemes, red), anti-CPN60 (apicoplast, red), anti-GRA5 (dense granules, red), anti-ARO (rhoptries, red), anti-HSP60 (mitochondria, red), anti-SAS6–2Ty or anti-Sfi1–2Ty (outer centrosome core, red), anti-CEP250-L1-2Ty (inner centrosome core, red), anti-kinetochore Nuf2–2Ty (kinetochore, red), anti-Chromo1–2Ty (centromere, red), and anti-RNG1–2Ty or anti-RNG2–2Ty (apical polar ring, red). Yellow arrows indicate the presence of remnant mother apical polar rings. Scale bar: 2 µm. (TIF) [file ppat.1013475.s002.tif]

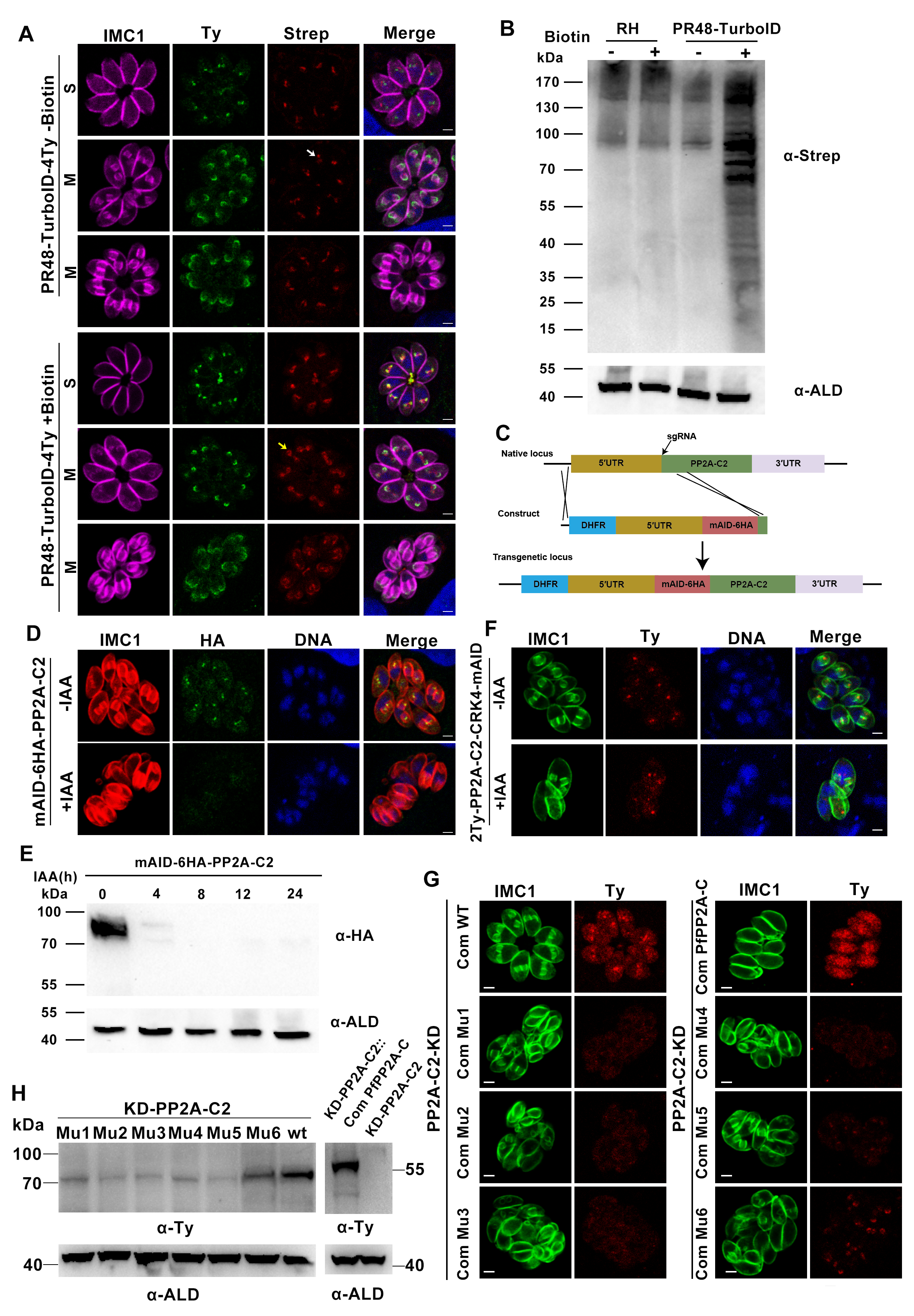

Supplement: S3 Fig — (A). Immunofluorescence analysis of the RH::PR48-TurboID-4Ty strain showing proteins in proximity to TgPR48 labeled and visualized with streptavidin conjugated with Alexa Fluor 594 after D-biotin treatment. Green: mouse anti-Ty; red: streptavidin Alexa Fluor 594 conjugate; magenta: rabbit anti-IMC1. Scale bar: 2 µm. (B). Western blot analysis confirming increased levels of streptavidin-HRP-labeled proteins in the RH::PR48-TurboID-4Ty strain after D-biotin induction. White arrow denotes biotinylated apicoplasts, and yellow arrow indicates proteins biotinylated by PR48-TurboID. Strep-HRP: Streptavidin-Horseradish Peroxidase conjugate; ALD: rabbit anti-aldolase. (C). Schematic representation of endogenous tagging at the N-terminus of the PP2A-C2 gene. Tag insertion was validated by PCR and DNA sequencing. (D). Immunofluorescence analysis of intracellular RH::mAID-6HA-PP2A-C2 parasites treated with or without IAA for 24 h. Parasites were stained with anti-IMC1 (red) and anti-HA (green) antibodies. Scale bar: 2 µm. (E). Western blot analysis of total protein extracts from RH::mAID-6HA-PP2A-C2 strains treated with IAA for varying durations. Protein degradation was confirmed with an anti-HA antibody, and ALD served as the loading control. (F). Immunofluorescence analysis showing the subcellular localization of 2Ty-PP2A-C2 in RH::CRK4-mAID parasites following treatment with or without IAA for 24 h, indicating that PP2A-C2 remained localized at the centrosome regardless of CRK4 depletion. Parasites were stained with anti-IMC1 (green) and anti-Ty (red) antibodies. Scale bar: 2 µm. (G). Functional analysis of the PP2A-C2 catalytic motif consensus sequences. Immunofluorescence analysis of PP2A-C2-depleted parasites re-expressing the wild-type or mutant versions of 2Ty-tagged PP2A-C2, or 2Ty-tagged PfPP2A-C under the control of native TgPP2A-C2 promoters. Parasites were cultured under normal conditions for 28 h, followed by immunofluorescence detection with anti-IMC1 (green) and [file ppat.1013475.s003.tif]

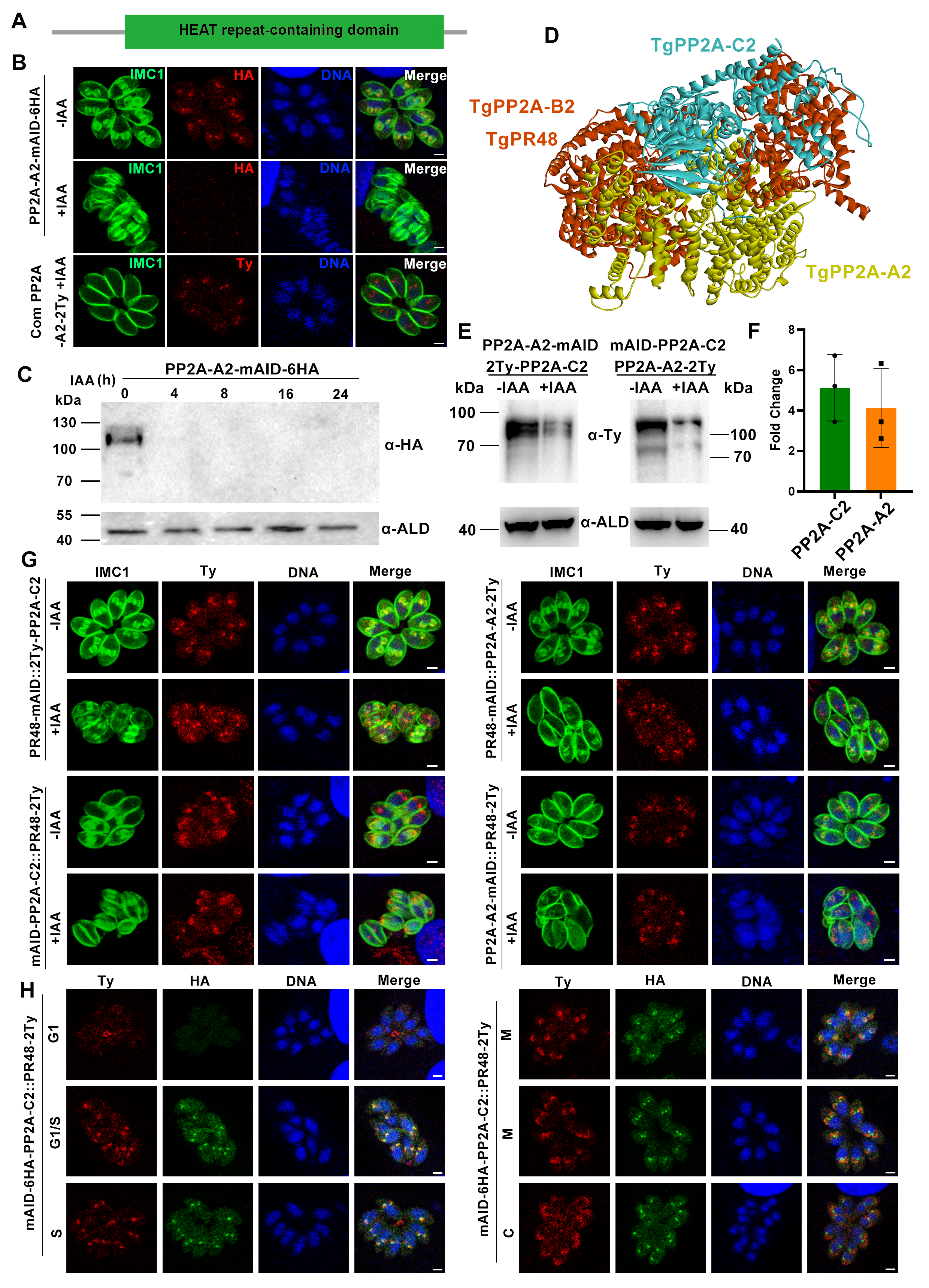

Supplement: S4 Fig — (A). Schematic representation of the PP2A-A2 gene model highlighting the HEAT repeat-containing domain. (B). Immunofluorescence analysis of intracellular RH::PP2A-A2-mAID-6HA and ComPP2A-A2-mAID-6HA parasites treated with IAA for 24 h. Parasites were stained with anti-IMC1 (green) and anti-HA (red) or anti-Ty (red) antibodies. Scale bar: 2 µm. (C). Western blot analysis of total protein extracts from the RH::PP2A-A2-mAID-6HA strains treated with IAA for varying durations. Degradation of the tagged protein was confirmed via the mAID system using an anti-HA antibody, with aldolase (ALD) as the loading control. (D). Structural model of the PP2A-2 holoenzyme generated using AlphaFold 3. (E). Western blot analysis of total protein extracts from the indicated strains treated with or without IAA for 24 h showed a reduction in one subunit when the other was depleted, indicating a functional link between PP2A-C2 and PP2A-A2. 2Ty-PP2A-C2 and PP2A-A2-2Ty proteins were detected using an anti-Ty antibody, with ALD serving as the loading control. (F). Quantification of fold changes in relative protein levels of PP2A-C2 in PP2A-A2-mAID parasites, and PP2A-A2 in mAID-PP2A-C2 parasites, was performed by comparing samples cultured in the absence or presence of IAA across three independent replicates. Western blotting band intensities were quantified using ImageJ software, and protein levels were normalized to the ALD signal, which served as a loading control. (G). Immunofluorescence analysis of the localization of TgPR48, PP2A-A2, and PP2A-C2 after depletion of mAID-tagged proteins. Strains were treated with IAA for 24 h to induce protein depletion. Ty-tagged proteins were detected using an anti-Ty antibody, and IMC1 was used for parasite visualization. Scale bar: 2 µm. (H). Immunofluorescence analysis of the co-localization of TgPR48 and PP2A-C2 during the tachyzoite cell cycle. RH::mAID-6HA-PP2A-C2::PR48–2Ty infected cells were fixed 24 h post-infection and stained with anti-HA ant [file ppat.1013475.s004.tif]

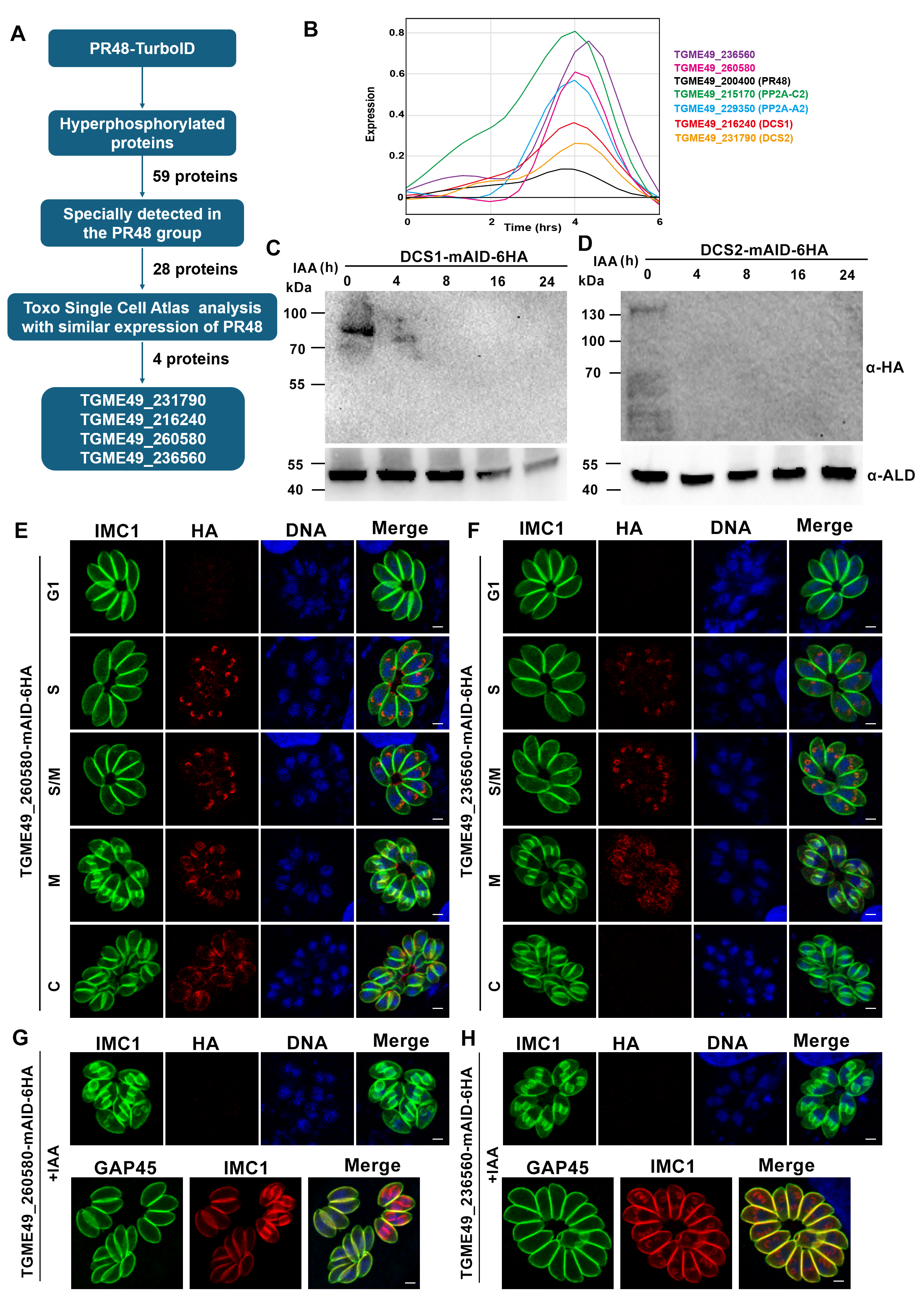

Supplement: S5 Fig — (A). Schematic representation illustrating the identification of the potential substrates of the PP2A-2 holoenzyme. (B). Expression patterns of the PP2A-2 holoenzyme and four hyperphosphorylated proteins based on data from the Toxo Single Cell Atlas. (C). Western blot analysis of total protein extracts from the RH::DCS1-mAID-6HA strains treated with IAA for different durations. Degradation of the tagged protein via the mAID system was confirmed using an anti-HA antibody. Aldolase (ALD) served as a loading control. (D). Western blot analysis of total protein extracts from the RH::DCS2-mAID-6HA strain treated with IAA for different durations. Degradation of the tagged protein via the mAID system was confirmed using an anti-HA antibody. ALD served as the loading control. (E). Immunofluorescence analysis of mAID-6HA-tagged TGME49_260580 during the tachyzoite cell cycle. RH::TGME49_260580-mAID-6HA-infected cells were fixed 24 h post-infection and stained with anti-IMC1 antibody (green) to visualize the cell cycle, anti-HA antibody (red) to detect TGME49_260580, and Hoechst dye (blue) to stain DNA. Scale bar: 2 µm. (F). Immunofluorescence analysis of mAID-6HA-tagged TGME49_236560 during the tachyzoite cell cycle. RH::TGME49_236560-mAID-6HA-infected cells were fixed 24 h post-infection and stained with anti-IMC1 antibody (green) to visualize the cell cycle, anti-HA antibody (red) to detect TGME49_236560, and Hoechst dye (blue) to stain DNA. Scale bar: 2 µm. (G) Immunofluorescence analysis of intracellular TGME49_260580-mAID-6HA parasites treated with IAA for 24–28 h. Parasites were stained with anti-IMC1 (green) and anti-HA (red) antibodies to detect TGME49_260580, and anti-GAP45 (green) and anti-IMC1 (red) antibodies to assess defects in daughter cell emergence. Scale bar: 2 µm. (H) Immunofluorescence analysis of intracellular TGME49_236560-mAID-6HA parasites treated with IAA for 24–28 h. Parasites were stained with anti-IMC1 (green) and anti-HA (red) antibodies to detect [file ppat.1013475.s005.tif]

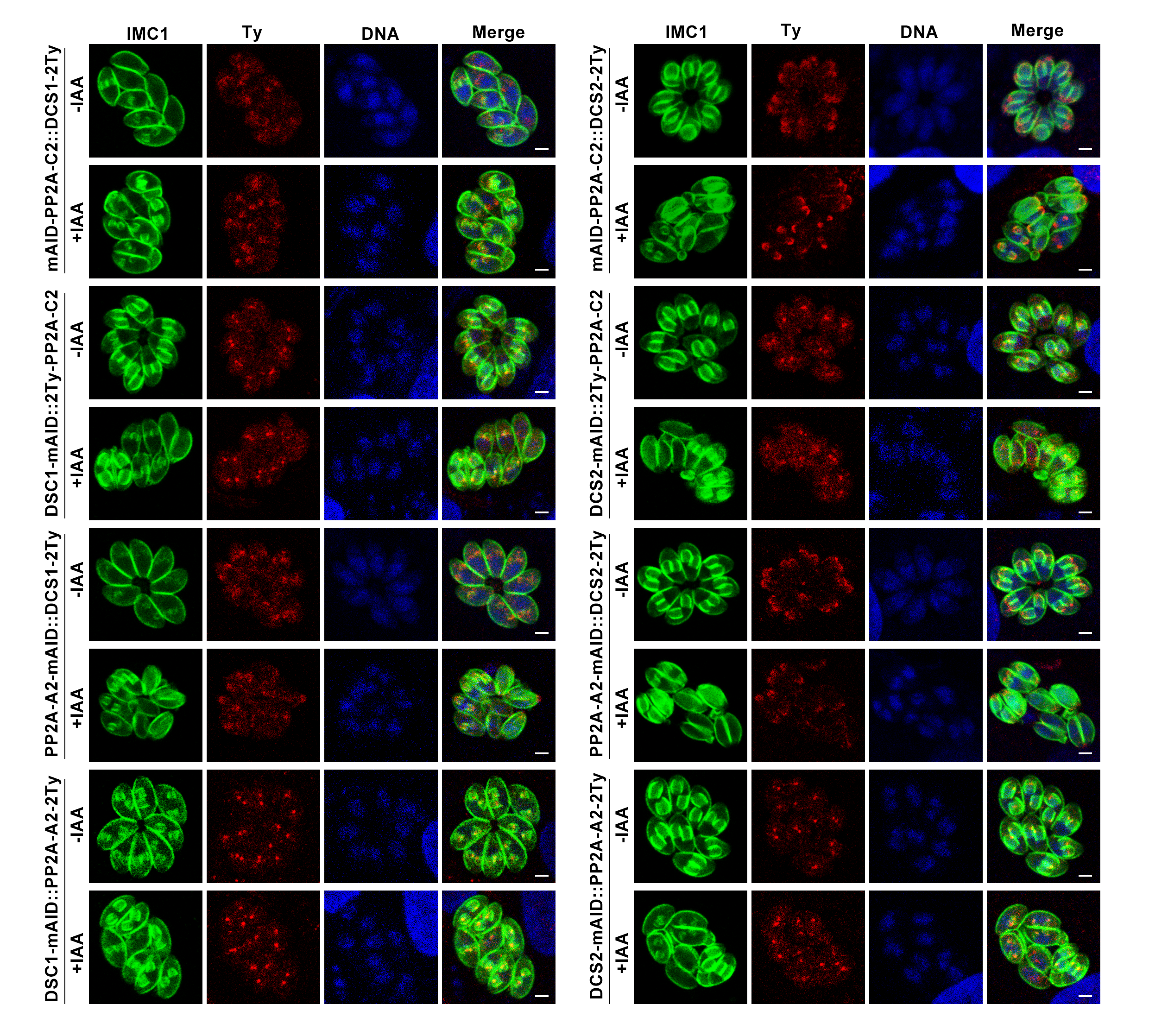

Supplement: S6 Fig — The indicated strains were treated with IAA for 24–28 h to induce depletion. Ty-tagged proteins were detected using an anti-Ty antibody, and rabbit anti-IMC1 was used to visualize the parasites. Scale bar: 2 µm. (TIF) [file ppat.1013475.s006.tif]

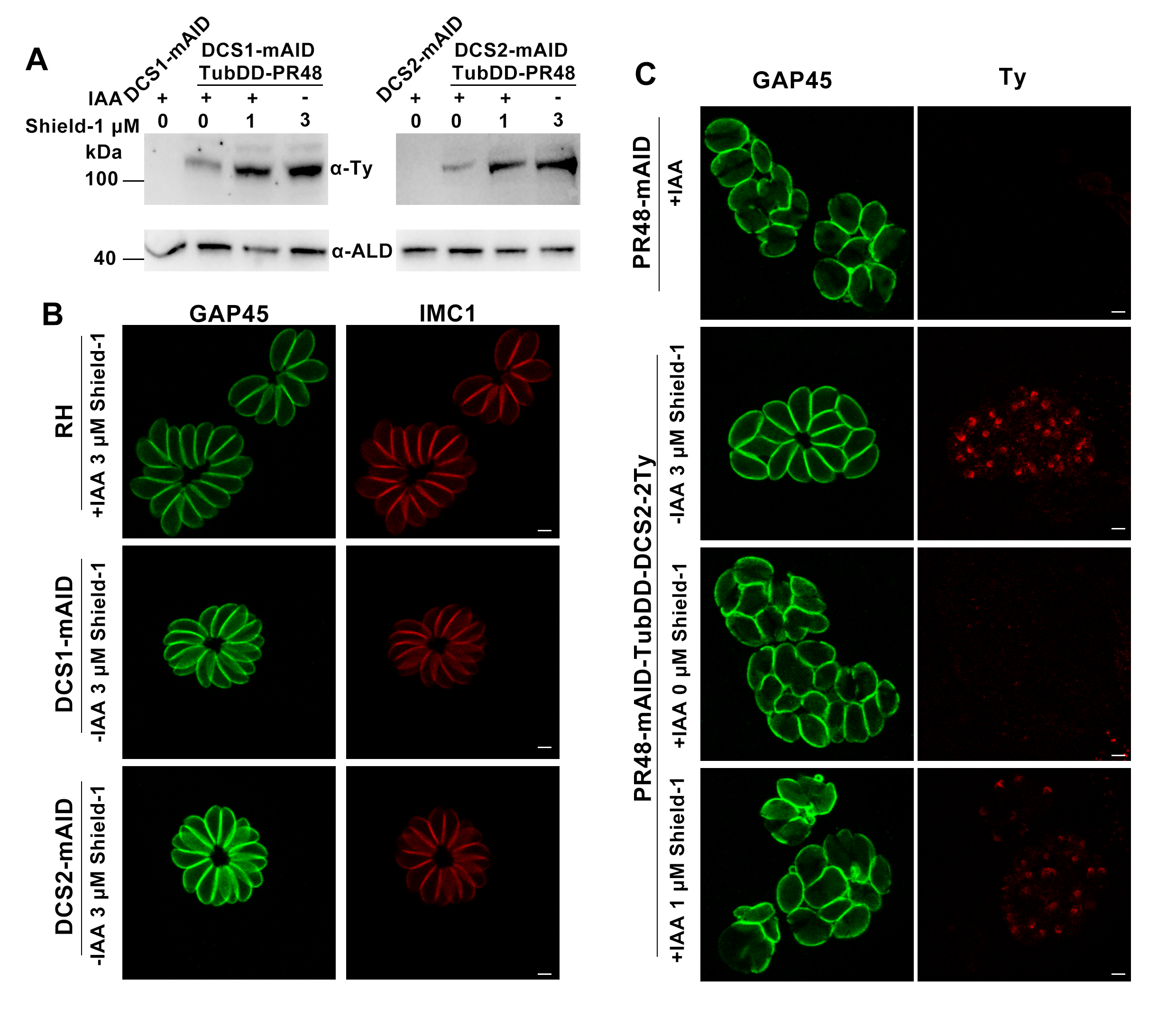

Supplement: S7 Fig — (A). Western blot analysis of total protein extracts from the indicated strains treated with IAA and/or Shield-1 for 48 h. The degradation domain system effectively regulates protein expression, as demonstrated by the levels of 2Ty-tagged TgPR48 detected using an anti-Ty antibody. Aldolase (ALD) was used as the loading control. (B). Immunofluorescence analysis of the indicated parasites to evaluate the effect of the Shield-1. Infected cells treated with IAA and/or Shield-1 were fixed 32 h post-infection and stained with anti-GAP45 (green) and anti-IMC1 (red) antibodies to assess daughter cell emergence. Scale bar: 2 µm. (C). Immunofluorescence analysis of the PR48-mAID-TubDD-DCS2–2Ty strain showing that overexpression of DCS2 cannot compensate for the depletion of TgPR48. Infected cells treated with IAA and/or Shield-1 were fixed 32 h post-infection and stained with anti-GAP45 (green) and anti-Ty (red) antibodies to evaluate daughter cell emergence. Scale bar: 2 µm. (TIF) [file ppat.1013475.s007.tif]
